# Effect of Mechanical Homogenization on Nopal Mucilage for the Treatment of a Real Cyanidation Barren Solution

**DOI:** 10.3390/gels12070569

**Published:** 2026-06-27

**Authors:** Allison Vianey Valle-Bravo, Brenda Hildeliza Camacho-Díaz, Denis Rodrigue, Glenda Pacheco-Vargas, Francisco Rodríguez-González, Isidra Guadalupe Ruiz-Martínez, Javier Solorza-Feria

**Affiliations:** 1 Centro de Desarrollo de Productos Bióticos, Instituto Politécnico Nacional, Yautepec C.P. 62739, Morelos, Mexico; avalleb2103@alumno.ipn.mx (A.V.V.-B.); bcamacho@ipn.mx (B.H.C.-D.); gpachechov@ipn.mx (G.P.-V.); frrodriguezg@ipn.mx (F.R.-G.); iruizm1500@alumno.ipn.mx (I.G.R.-M.); 2 Department of Chemical Engineering, Université Laval, Quebec City, QC G1V 0A6, Canada

**Keywords:** *Opuntia ficus-indica* mucilage, mechanical homogenization, barren solution treatment

## Abstract

This study investigated the effect of brief mechanical homogenization using a household blender on the properties of nopal mucilage and its performance in removing potentially toxic elements (PTEs), specifically Pb, Ni, As, Cd, and Zn, from a real cyanidation barren solution. An aqueous extract from *Opuntia ficus-indica* cladodes was homogenized for 0, 30, or 60 s before spray drying, yielding powders designated as CA, CB, and CC. The powders and water-reconstituted dispersions were characterized and evaluated in coagulation–flocculation assays. Homogenization reduced water activity and average hydrodynamic diameter and significantly modified the ζ potential, although the effects were not proportional to processing time. At 10% *w·v*^−1^, the reconstituted mucilages showed frequency-dependent viscoelastic behavior consistent with a transient gel-like organization. All treatments removed more than 98% of Pb, Ni, and As at doses of 200–800 mg·L^−1^. Cd removal was more variable and significantly affected by mucilage type, whereas Zn showed lower, non-monotonic removal. ESEM–EDS detected PTE-bearing inorganic domains within the recovered flocs, corroborating transfer from the liquid to the solid phase. Overall, mechanical homogenization modified the colloidal, supramolecular, and gel-related properties of spray-dried nopal mucilage, which showed potential as a multifunctional hydrocolloid for treating chemically complex cyanidation process streams.

## 1. Introduction

Nopal comprises most species of the genus *Opuntia* [1]. Among them, *Opuntia ficus-indica* is widely cultivated in arid and semiarid regions. Its cladodes are water-rich tissues whose water-retention capacity is partly associated with the presence of mucilage [2]. This mucilage is a heteropolysaccharide composed mainly of arabinose, xylose, galactose, and variable proportions of galacturonic acid [3,4]. Owing to its hydrocolloid nature, nopal mucilage forms viscous aqueous dispersions and can develop gel-like structures depending on factors such as concentration, pH, and Ca^2+^ availability [2,5].

The functional properties of nopal mucilage depend on its chemical composition and macromolecular organization, both of which may vary according to the extraction method and processing conditions [2,6]. In conventional protocols, mechanical grinding or blending of fresh or dried cladodes is commonly used to reduce plant-tissue size, increase the contact area during aqueous maceration, and promote mucilage release. However, a substantial portion of the literature does not report the grinding, blending, or homogenization time applied during this stage [3,7,8,9,10]. In studies that report this variable, processing times in commercial blenders vary considerably, including 45–50 s at maximum speed [11] and 1 min [12,13]. Moreover, others mention grinding without specifying the applied conditions [14,15].

This lack of methodological standardization is relevant because mechanical homogenization is not necessarily a neutral step for polymer solutions. In systems exposed to hydrodynamic fields, shear forces can modify polymer conformation, colloidal organization, and rheological behavior. Harrington [16] proposed that, in high-speed rotary homogenizers, the formation of a boundary layer near the blade generates velocity gradients capable of introducing tensile stresses on polymer chains. Furthermore, Wang et al. [17] reported that homogenization can generate high shear stresses and inertial forces and observed that increasing homogenization pressure reduced the storage modulus (G′) and loss modulus (G″) of flaxseed gum solutions. These findings suggest that shear history can be important for interpreting the properties of reconstituted mucilages, particularly when processing, structure, and functional performance are intended to be linked.

This relationship is especially important because nopal mucilage has been studied as a thickener, a gelling agent, an encapsulating material, and an environmental remediation agent [6]. Within the latter field, its use as a natural coagulant and flocculant has received attention because of its ability to promote the removal of contaminants, including potentially toxic elements (PTEs), such as the metals Pb, Ni, Cd, and Zn and the metalloid As, through mechanisms involving adsorption, charge-related interactions, polymer bridging, and physical entrapment [18,19]. However, the performance of these biopolymers has rarely been evaluated while considering their processing history. Such processing may alter their physicochemical, colloidal, rheological, and thermal properties and, consequently, their interactions with PTEs present in dissolved, colloidal, or particulate forms.

Gold mining is an economically relevant sector in Mexico [20] and generates cyanide-bearing process solutions during gold and silver extraction. After precious-metal recovery, the barren solution (BS) may retain residual cyanide species and PTEs [21]; therefore, these process streams require treatment to reduce their contaminant load before disposal or recirculation [22]. Depending on their concentration, chemical form, mobility, and exposure pathway, PTEs may pose risks to aquatic ecosystems and human health [23]. Although methods such as chemical precipitation, ion exchange, and adsorption can be effective, their practical implementation may be limited by reagent and treatment costs, sludge generation, and specialized operational requirements [24,25]. Previous studies have demonstrated the potential of nopal mucilage for PTE removal in synthetic and natural aqueous systems [6,26].

As a gel material, nopal mucilage offers several potential advantages, including biodegradability, availability from renewable plant biomass, aqueous processability, and the capacity to assist in the removal of elements such as Pb, Cd, As, Cr, and Cu [6], and produces less sludge than synthetic coagulants [19]. However, challenges include batch-to-batch variability depending on irrigation [2] and sensitivity to pH, ionic strength, and extraction method [6]. Understanding these pros and cons helps evaluate its real potential as a gel-based coagulant.

Despite the growing interest in nopal mucilage as a bio-based alternative for water treatment, it remains unclear how the duration of mechanical homogenization using a widely available household blender before spray drying affects the properties of the reconstituted mucilage and its performance as a natural coagulant–flocculant for removing PTEs from a real BS. Accordingly, this study was conceived as an initial proof of concept to evaluate the effects of mechanical homogenization on the physicochemical, colloidal, thermal, microstructural, and rheological properties of spray-dried nopal mucilage and its treatment performance in this chemically complex industrial process stream.

## 2. Results and Discussion

### 2.1. Physicochemical and Colloidal Properties

To facilitate interpretation of the results, the samples were identified according to the homogenization time applied to the aqueous mucilage extract before spray drying. CA corresponded to the non-homogenized control, whereas CB and CC corresponded to extracts homogenized for 30 and 60 s, respectively. After this process, the three extracts were spray-dried to obtain mucilage powders. Unless otherwise stated, CA, CB, and CC refer to these powders. When the analyses required prior hydration, the use of water-reconstituted mucilages is explicitly indicated.

The proximate composition and water activity of CA, CB, and CC are summarized in Table 1. Moisture content did not differ significantly among samples (*p* > 0.05), which can be attributed to the fact that all samples were spray-dried under identical conditions. In contrast, water activity (a_w_), measured at ambient temperature (36 ± 1 °C), decreased significantly after homogenization, from 0.135 in CA to 0.110 in CB and 0.115 in CC (*p* < 0.05). However, no significant differences were observed between CB and CC, indicating that the effect occurred within the first 30 s of homogenization and that extending the process to 60 s did not further reduce a_w_. This decrease, without changes in total moisture content, suggests a modification in the way water is associated within the polysaccharide matrix. In this regard, Quintero-García et al. [8], although they did not evaluate water activity, also reported that mechanical processing of fresh cladodes by wet milling with a blade mill did not significantly modify the moisture content of nopal mucilage.

Protein and ash contents were significantly higher in CC than in CA and CB. These differences are unlikely to derive from mechanical homogenization itself, since this process should not generate or eliminate proteins or minerals. A more plausible explanation is the natural variability of the plant material. Although the cladodes were harvested on a single day and from the same field, nopal composition can vary among cladodes due to physiological, edaphic, and developmental factors [2]. In addition, the harvested batch was subdivided for aqueous mucilage extraction, which may have contributed to small compositional differences among samples.

Particle size and ζ potential were analyzed in CA, CB, and CC mucilages reconstituted in water at 0.13% *w·v*^−1^ and at their native pH of 6.8 ± 0.2. The ζ potential differed significantly among the three samples (*p* < 0.05). Homogenization made the ζ potential more negative relative to the control, shifting from −15.7 mV in CA to −18.3 mV in CB and −17.0 mV in CC. CB showed the most negative value, followed by CC and CA, indicating that homogenization modified the surface charge of the reconstituted mucilage and favored greater relative electrostatic repulsion, which is associated with higher colloidal stability [28]. However, the fact that CC was less negative than CB suggests that the effect of homogenization time on surface charge was not linear, as observed in other hydrocolloids subjected to mechanical shear [17].

The average particle diameter decreased significantly from 1.8 μm in CA to 1.3 μm in CB and CC (*p* < 0.05). CA differed from CB and CC, whereas no significant differences were observed between the homogenized samples. This reduction indicates that homogenization favored the fragmentation or dispersion of colloidal structures present in the reconstituted mucilage. However, the absence of differences between CB and CC suggests the existence of a size reduction threshold within the first 30 s of homogenization, beyond which additional shear did not further decrease the average diameter. Despite this reduction in particle size, the polydispersity index (PDI) remained around 0.6 in all samples, without significant differences. This indicates that homogenization modified the average particle size but did not significantly narrow the size distribution. A PDI above 0.5 is commonly associated with a broad distribution [5], which is consistent with the inherent polydispersity of complex colloidal systems such as reconstituted nopal mucilage. These results are compatible with previous studies on the mechanical homogenization of flaxseed gum and chia mucilage, in which shear modified particle size and colloidal properties without necessarily producing linear responses as the intensity or duration of mechanical treatment increased [17,29].

### 2.2. Rheological Behavior of Reconstituted Nopal Mucilage

**Figure 1:**
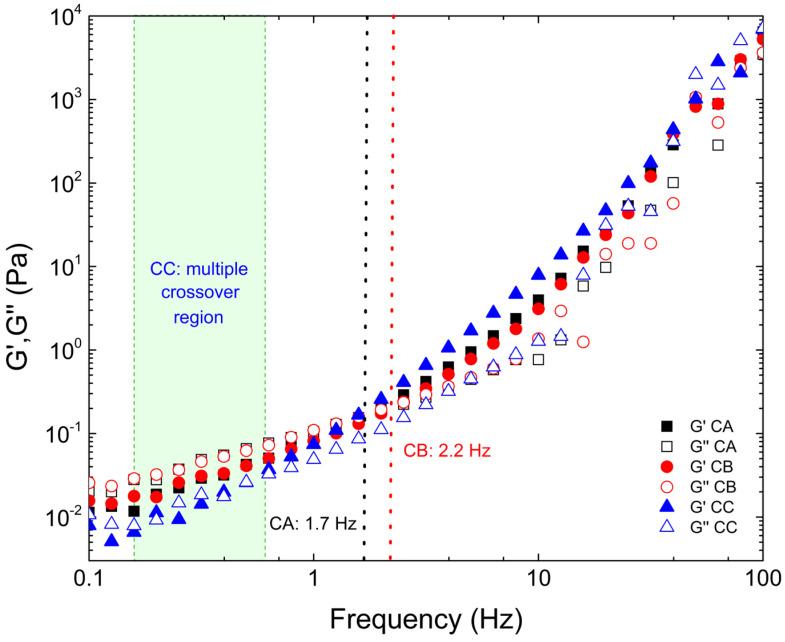
Frequency sweeps of the storage modulus (G′, filled symbols) and loss modulus (G″, open symbols) of reconstituted nopal mucilage dispersions prepared from powders obtained at different homogenization times. CA corresponds to the non-homogenized control, CB to mucilage homogenized for 30 s, and CC to mucilage homogenized for 60 s. Measurements were performed at 20 °C and 0.5% strain within the linear viscoelastic region. The vertical dotted line indicates the G′ and G″ crossover frequency for CA at approximately 1.7 Hz. The crossover for CB occurs at approximately 2.2 Hz. CC shows multiple crossovers at low frequencies, highlighted by the shaded region.

The rheological properties of CA, CB, and CC mucilages reconstituted at 10% *w·v*^−1^ showed that mechanical homogenization modified the structural organization of the system, although the effect did not follow a linear relationship with processing time. In the frequency sweeps performed within the linear viscoelastic region (Figure 1), the samples showed a frequency-dependent response. At low frequencies, the viscous modulus (G″) was higher than the elastic modulus (G′) over part of the interval, whereas increasing frequency led to a transition toward G′ predominance. This behavior is compatible with systems in which polymer chains, in the state of random coils, interact mainly through physical entanglements rather than through a permanently crosslinked network, corresponding to a transient entanglement network [30].

In CA and CB, crossover points between G′ and G″ were identified around 1.7 and 2.2 Hz, respectively. Above these frequencies, G′ remained higher than G″, indicating that the system showed a greater elastic contribution when less time was available for relaxation during oscillatory deformation. This transition suggests that the structures present in the mucilage can reorganize at low frequencies but offer greater elastic resistance when deformation occurs more rapidly. In CC, the multiple crossover points observed at low frequencies suggest a less defined structural response, possibly associated with transient physical associations, heterogeneous hydrated domains, or overlapping relaxation processes [31].

The rheological response of mucilage should be interpreted by considering concentration, pH, ionic strength, processing history, and reconstitution state. Although this study used concentrated dispersions at 10% *w·v*^−1^ to obtain robust signals under the operating conditions of the rheometer, the observed behavior is similar to that reported by Quinzio et al. [31] at concentrations of 0.5% and 1%, where viscous predominance at low frequencies followed by elastic predominance at higher frequencies was associated with an entanglement network. In contrast, at concentrations of 1.5% and 4.5%, these authors observed that G′ exceeded G″ throughout the entire frequency range, which was interpreted as a tendency to form macromolecular networks with substantial elastic properties, similar to the more rigid and structured behavior of xanthan gum. This comparison suggests that the nominal concentration of mucilage does not by itself determine effective structural connectivity, since previous processing, colloidal dispersion, and chain reorganization can also modulate the viscoelastic response.

**Figure 2:**
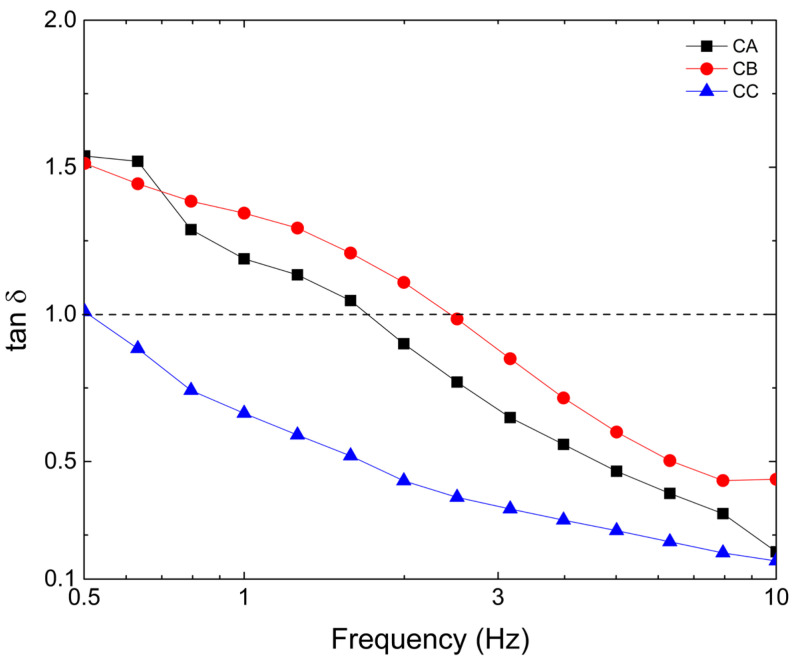
Loss tangent (tan δ = G″/G′) as a function of frequency for reconstituted nopal mucilage dispersions prepared from CA, CB, and CC powders. CA corresponds to the non-homogenized control, CB to mucilage homogenized for 30 s, and CC to mucilage homogenized for 60 s. The horizontal dashed line indicates tan δ = 1, which marks the transition between predominant viscous behavior (tan δ > 1) and predominant elastic behavior (tan δ < 1).

Comparison with other studies confirms that mucilage can exhibit contrasting rheological responses depending on formulation and analytical conditions. Torregrossa et al. [5], when formulating mucilage with sucrose and calcium salts, reported that G″ consistently exceeded G′ over an angular frequency range of 0.1 to 100 rad s^−1^, equivalent to approximately 0.016 to 15.9 Hz, indicating a predominantly viscous response even in weak hydrogels. Contreras-Padilla et al. [7], in turn, analyzed 1% aqueous mucilage suspensions over an angular frequency range of 1 to 600 rad s^−1^, equivalent to approximately 0.159 to 95.5 Hz, and reported that G′ and G″ remained close to each other, with predominance of viscous behavior. Together, these findings place the present results in an intermediate region, since the reconstituted mucilages did not behave as strong gels throughout the entire frequency range but developed a more elastic, hydrogel-type response at higher frequencies.

tan δ curves (Figure 2) reinforced this interpretation. Since tan δ represents the G″/G′ ratio, values above 1 indicate viscous predominance, whereas values below 1 indicate elastic predominance [30]. In CA, tan δ shifted from values above 1 to values below 1 between 1.585 and 1.995 Hz, whereas in CB this transition occurred between 1.995 and 2.512 Hz. In CC, the transition occurred at lower frequencies, between 0.5012 and 0.631 Hz. These results confirm that the three samples shifted toward a more elastic response as frequency increased, albeit with different transition scales. CB showed the highest tan δ values over the evaluated interval and maintained a value of 0.44 at 10 Hz, higher than those of CA (0.19) and CC (0.16). This indicates that CB retained a greater relative viscous contribution, even when G′ predominated at high frequencies.

The greater relative viscous contribution of CB is consistent with its more negative ζ potential and reduced average particle size. Greater electrostatic repulsion may favor a more stable colloidal dispersion, with fewer effective contacts among chains or hydrated domains. Under this scenario, the structures may move or slide more independently during oscillatory deformation, increasing relative viscous dissipation and delaying the transition toward elastic predominance compared with CA and CC.

**Figure 3:**
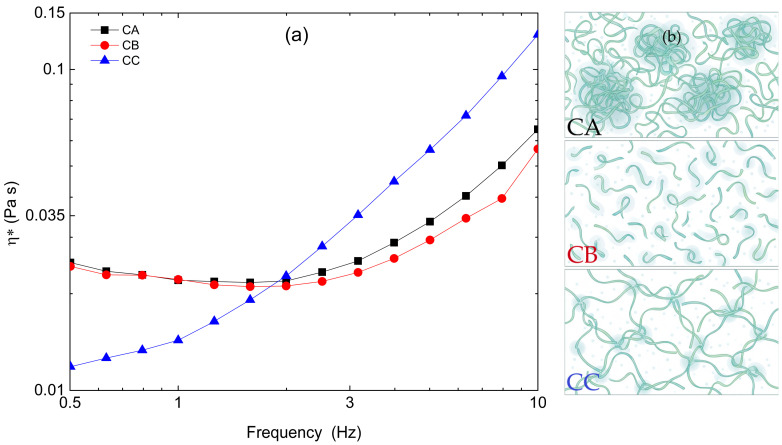
Rheological response and proposed structural interpretation of reconstituted nopal mucilage dispersions. (**a**) Complex viscosity (η*) as a function of frequency for CA, CB, and CC. The CA sample corresponds to the non-homogenized control, CB to mucilage homogenized for 30 s, and CC to mucilage homogenized for 60 s. (**b**) Schematic representation of the proposed structural organization of the reconstituted mucilages, where CA is represented by larger hydrated aggregates, CB by a more dispersed and electrostatically stabilized structure, and CC by a weak, discontinuous network with transient physical associations.

The complex viscosity (η*) (Figure 3a) also showed relevant differences among samples. Between 0.5 and 10 Hz, the viscosity of CA increased from 0.0250 to 0.0651 Pa·s, whereas CB increased from 0.0243 to 0.0566 Pa·s. Although both samples showed an increase in η* with frequency, their values remained close to each other and the increase was moderate. In contrast, CC increased from 0.0119 to 0.1284 Pa·s, indicating a much greater frequency-dependent response. This behavior should not be interpreted as shear thickening under steady flow, but rather as greater dynamic resistance under oscillatory deformation. In this sense, the marked increase in η* in CC suggests that its viscoelastic structure responded with a substantial increase in the complex modulus (G*) as the time available for structural relaxation decreased.

These results can be related to physicochemical and colloidal characterization. CA retained the largest average particle diameter and the least negative ζ potential, suggesting the presence of larger and less dispersed hydrated domains. CB showed a smaller average size and the most negative ζ potential, indicating greater colloidal dispersion and stronger electrostatic repulsion. This condition may have limited the formation of structural contacts among chains, explaining its greater relative viscous contribution. In CC, the average size did not decrease further compared with CB, but the ζ potential was less negative and η* increased markedly with frequency. This suggests that increasing homogenization time in the aqueous nopal mucilage extract did not produce additional fragmentation of the system, but rather a possible partial reorganization of chains or hydrated domains.

In general, the data suggest that homogenization for 30 s mainly favored system dispersion, whereas homogenization for 60 s may have induced partial structural reorganization, with the formation of weak and transient physical associations. Under oscillatory deformation, these associations could break and reform at low frequencies, generating a less defined response, whereas at higher frequencies they would not fully relax and would contribute to greater dynamic resistance. Therefore, the reconstituted mucilages should not be described as strong gels, but as frequency-dependent viscoelastic systems with a transition toward hydrogel type behavior at higher frequencies.

Figure 3b schematically summarizes this interpretation. CA is represented as a system with larger hydrated aggregates, CB as a more dispersed and electrostatically stabilized structure, and CC as a weak and discontinuous network with transient associations that could explain its greater frequency dependence during oscillatory deformation.

### 2.3. Fourier Transform Infrared Spectroscopy (FTIR) of Nopal Mucilage

**Figure 4:**
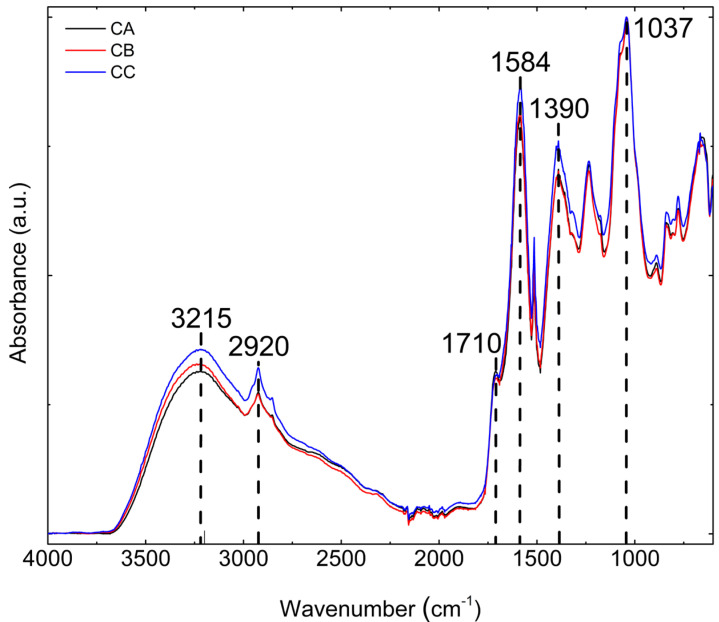
Normalized ATR-FTIR spectra of spray-dried nopal mucilage powders obtained at different homogenization times. CA corresponds to the non-homogenized control, CB to mucilage homogenized for 30 s, and CC to mucilage homogenized for 60 s. Spectra were recorded over the range of 4000–400 cm^−1^ and normalized to the 0–1 interval.

The molecular structure of CA, CB, and CC mucilage powders were analyzed by ATR-FTIR spectroscopy. Figure 4 shows the normalized spectra in the 4000 to 400 cm^−1^ range. Overall, the three samples showed the characteristic profile spectra of nopal mucilage polysaccharides, with bands associated with hydroxyl groups, C-H bonds, carbonyl and carboxylate groups, and vibrations characteristic of the glycosidic backbone.

The broad band centered around 3215 cm^−1^ is mainly attributed to O-H stretching vibrations, related to intra- and intermolecular hydrogen bonding of retained water and carbohydrates within the polysaccharide matrix [7,8]. The signal observed at 2920 cm^−1^ corresponds to C-H stretching vibrations associated with methylene groups and pyranose ring structures [7,8,32]. The band near 1710 cm^−1^ can be related to C=O stretching vibrations of carboxylic or carbonyl groups present in uronic acids, whereas the signal around 1584 cm^−1^ is mainly associated with asymmetric stretching of the carboxylate group COO^−^, characteristic of galacturonic acid residues and other uronic acids [7,8,32]. The band at 1390 cm^−1^ is attributed to symmetric stretching of the carboxylate group COO^−^ and to contributions from O-H bending vibrations [8,32]. Finally, the intense signal at 1037 cm^−1^ corresponds to C-O and C-C stretching vibrations of the sugar backbone, a characteristic region of polysaccharides [7,8,32].

Although the spectra of CA, CB, and CC retained the same overall pattern, relative intensity changes were observed in specific regions. In particular, the bands at 3215, 2920, and 1390 cm^−1^ progressively increased with homogenization time. Since the spectra were normalized, these changes should be interpreted as relative variations in the spectral contribution of specific functional groups, rather than as an absolute increase in their concentration. Even so, the increase in intensity in the O-H region suggests that homogenization modified the environment of hydroxyl groups and the organization of hydrogen bonds within the polysaccharide matrix. Similarly, the changes at 2920 and 1390 cm^−1^ indicate modifications in the vibrational environment of C-H and carboxylate groups, respectively.

These results are compatible with the hypothesis that the mechanical shear applied during homogenization may have altered the conformation and supramolecular arrangement of the mucilage, favoring the relative exposure of hydroxyl and carboxylate groups. However, these changes should not be interpreted as direct evidence of chain scission, since complementary information on molar mass or molecular weight distribution would be necessary to confirm macromolecular degradation. In this regard, previous studies have shown that homogenization can modify the structural, colloidal, and rheological properties of polysaccharide hydrocolloids [17], as well as induce spectral changes in regions associated with uronic acids, as reported for chia mucilage treated by high-pressure homogenization [29].

### 2.4. Thermogravimetric Analysis (TGA) of Nopal Mucilages

**Figure 5:**
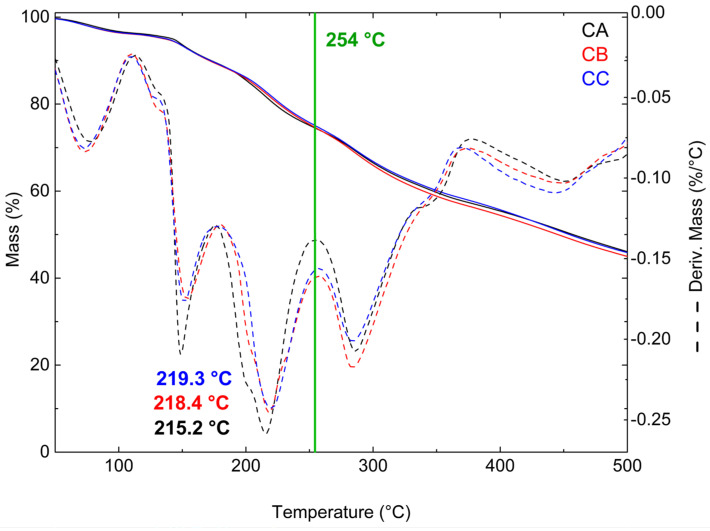
Thermogravimetric analysis (TGA) and derivative thermogravimetry (DTG) curves of spray-dried nopal mucilage powders. CA corresponds to the non-homogenized control, CB to mucilage homogenized for 30 s, and CC to mucilage homogenized for 60 s. The green vertical line indicates the Tmax of 254 °C reported for mucilage extracted from fresh cladodes [8]. The numerical labels in black, red, and blue indicate the Tmax values obtained in this study for CA, CB, and CC, respectively.

The thermal stability of CA, CB, and CC mucilage powders was evaluated by TGA and DTG. Figure 5 shows the thermal profiles, and Table 2 summarizes the main parameters obtained. The initial mass loss occurred mainly below 200 °C and is attributed to the evaporation of free water, adsorbed water, and water associated with the polysaccharide matrix through hydrogen bonding [8,32,33]. The temperature corresponding to 5% mass loss was 142.2 °C for CA, 136.1 °C for CB, and 137.6 °C for CC, whereas 10% mass loss was reached at 172.6, 171.3, and 172.3 °C, respectively. These differences were small, suggesting that homogenization only slightly modified the initial water removal stage without substantially altering the overall thermal behavior of the powders.

The main degradation event occurred between 215 and 220 °C and is attributed to the thermal decomposition of the polysaccharide structure, including dehydration, depolymerization, and rupture of components associated with the pectic fraction and uronic acids. This range agrees with previous reports for *Opuntia* mucilages, in which the major mass loss occurs approximately between 200 and 320 °C [8,32]. CA showed a Tmax of 215.2 °C, whereas CB and CC showed slightly higher values of 218.4 and 219.3 °C. Because these differences were below 5 °C, the results indicate that homogenization did not significantly compromise the main thermal stability of the mucilage.

However, the interpretation of CC should consider its higher ash content. The mineral fraction may influence water retention, interactions with carboxylate groups, and the apparent thermal stability of the polysaccharide matrix. Therefore, the slight increase in Tmax observed for CC should not be attributed exclusively to the longer homogenization time, but rather to the combined effect of shear history, physical organization of the mucilage, and the contribution of the inorganic fraction [6,8].

The maximum degradation rate decreased slightly after homogenization. DTGmax changed from −0.2587%/°C in CA to −0.2455%/°C in CB and −0.2425%/°C in CC, indicating a slightly slower maximum thermal degradation rate in the homogenized samples. In the region after the main peak, between 280 and 330 °C, CB showed more negative DTG values than CA and CC. At 284 °C, the values were −0.2176%/°C for CB, −0.2057%/°C for CA, and −0.2012%/°C for CC. This difference suggests that homogenization for 30 s may have modified the thermal stability of a minor material fraction, possibly through greater dispersion or accessibility of polysaccharide domains, whereas in CC this effect may have been partially offset by structural reorganization and by its higher mineral content.

In this study, Tmax values ranged from 215.2 to 219.3 °C, below the Tmax of 254 °C reported by Quintero-García et al. [8] for mucilage extracted from fresh cladodes. This difference should not be interpreted solely as a consequence of homogenization, since mucilage thermal stability may depend on multiple factors, including the origin of the plant material, cladode age or developmental stage, extraction method, drying process, mineral composition, and macromolecular characteristics of the polysaccharide. In this regard, Rodríguez-González et al. [4] noted that growing conditions may influence polymer molecular weight, which is relevant because chain size and macromolecular organization can affect the thermal response of mucilage.

Overall, the TGA and DTG results indicate that the mucilage powders retained adequate thermal stability after homogenization and spray drying. The observed changes were subtle and were mainly reflected in the initial mass loss stage, the maximum degradation rate, and the region after the main event, but not in a relevant shift of Tmax.

### 2.5. Microstructural Analysis of Mucilage Powders

**Figure 6:**
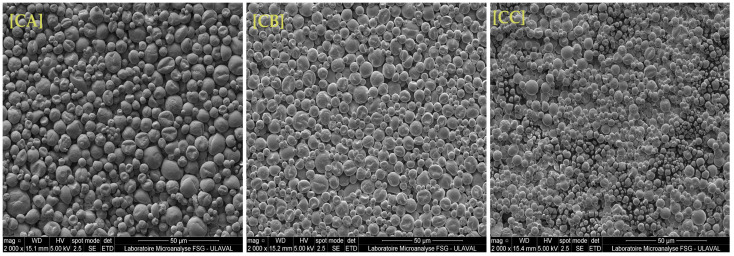
Scanning electron microscopy (SEM) images of the surface morphology of spray-dried nopal mucilage powders obtained at different homogenization times. CA: non-homogenized control. CB: mucilage homogenized for 30 s. CC: mucilage homogenized for 60 s. Images were acquired at 2000× magnification.

The surface microstructure of the spray-dried mucilage powders was examined by scanning electron microscopy (SEM). Figure 6 shows representative micrographs of CA, CB, and CC, corresponding to the non-homogenized control and to the mucilages homogenized for 30 and 60 s, respectively. The images were quantitatively analyzed using texture descriptors derived from the gray level co-occurrence matrix (GLCM) and fractal dimension (FD) analysis, tools commonly used to characterize morphological and textural changes in processed biopolymeric systems [34,35]. Table 3 summarizes the angular second moment (ASM), contrast, inverse difference moment (IDM), entropy, and total fractal dimension of the three samples.

ASM, which is associated with textural uniformity, increased significantly (*p* < 0.05) from 5.00 × 10^−4^ in CA to 7.47 × 10^−4^ in CB and 7.77 × 10^−4^ in CC, without additional significant differences between the homogenized samples. This behavior indicates that mechanical homogenization applied to the aqueous extract before spray drying favored the formation of more uniform surfaces. Consistently, contrast decreased from 469.17 in CA to 375.56 in CB and 353.08 in CC, suggesting a reduction in local intensity variations and, therefore, lower surface heterogeneity.

Fractal dimension decreased slightly but significantly from 2.23 in CA to 2.16 in CB and 2.17 in CC. In surfaces analyzed by fractal dimension, values closer to 2 are associated with relatively less complex surfaces, whereas values approaching 3 indicate greater roughness and geometric complexity [36,37]. Therefore, the observed decrease in FD suggests that homogenization reduced the surface complexity of the spray-dried particles. This result is consistent with the reduction in the average hydrodynamic diameter of the reconstituted mucilages, which decreased from 1.8 μm in CA to 1.3 μm in CB and CC (Table 1), as well as with the FTIR changes (Figure 4), where relative variations in bands associated with hydroxyl and carboxylate groups suggest modifications in the chemical and supramolecular environment of the mucilage.

Overall, the GLCM descriptors and fractal dimension indicate that homogenization produced powders with more uniform, less heterogeneous, and geometrically less complex surfaces. The fact that CB and CC showed statistically similar values for texture parameters and FD suggests that 30 s of homogenization were sufficient to reach a plateau in the surface changes detectable by this analysis, without evident additional modifications after extending the process to 60 s. This trend agrees with the behavior observed in particle size and in some thermal parameters, where CB and CC also showed similar responses. Therefore, the microstructural results support the general interpretation that homogenization mainly modified the physical and supramolecular arrangement of the mucilage, without producing a linear progression of the effect as processing time increased.

### 2.6. Removal of PTEs Using Reconstituted Nopal Mucilages

The data in Table 4 showed that reconstituted CA, CB, and CC mucilages effectively decreased the concentrations of Pb, Ni, and As remaining in the liquid phase of the real BS. Removal efficiencies exceeded 98% under all evaluated conditions, and the two-way ANOVA showed no significant effects of mucilage type, dose, or their interaction for these elements (*p* > 0.05). Increasing the mucilage concentration from 200 to 800 mg·L^−1^ therefore produced no statistically detectable improvement. Within the evaluated range, 200 mg·L^−1^ was sufficient to attain the observed removal plateau, although lower concentrations should be investigated before defining a minimum effective dose.

The small numerical differences among treatments, particularly for As, should be interpreted cautiously because several residual concentrations were close to or below the corresponding analytical limits. Under these conditions, removal values approaching 100% indicate a marked decrease in the concentration remaining in the liquid phase but do not necessarily represent analytically distinguishable differences among treatments. Moreover, because removal was calculated from this decrease, the results do not independently distinguish between adsorption, precipitation, capture of colloidal or suspended phases, incorporation into flocs, and sedimentation.

A plausible mechanistic framework begins with the uronic acid-rich fraction of nopal mucilage. At the native pH of the barren solution, 11.28, uronic acid carboxyl groups would be expected to occur predominantly in their deprotonated form, COO^−^, providing negatively charged domains along the polysaccharide chains. González-Avilez et al. [26] proposed that the increased negative charge of Opuntia mucilage under basic conditions favors interactions with accessible positively charged element species. In addition to electrostatic attraction, carboxylate oxygen atoms may participate in coordination or ion-exchange interactions. Ibarra-Rodríguez et al. [38] similarly related the retention of metallic ions by purified nopal pectin to carboxylate- and hydroxyl-containing domains associated with galacturonic acid.

Direct interaction with dissolved cationic species is unlikely to account for the complete response of this chemically complex matrix. Whole nopal mucilage may also adsorb onto inorganic or colloidal surfaces and connect particles through polymer bridging. González-Avilez et al. [26] proposed that removal from natural water may involve both interactions with oppositely charged species and bridging between mucilage chains and suspended particles. Likewise, pH, surface charge, chemical affinity, and polyelectrolyte dosage are recognized as important factors governing particle destabilization and separation during coagulation–flocculation [39]. The results are therefore consistent with two complementary pathways: interaction of accessible element species with deprotonated uronic acid domains and capture of precipitated, mineral-associated, or colloidal fractions through polymer adsorption, bridging, floc formation, and sedimentation. Their individual contributions were not quantified separately.

The colloidal characterization provides additional context for this interpretation. Homogenization reduced the average particle diameter from 1.8 µm in CA to 1.3 µm in CB and CC, while CB showed the most negative ζ potential. However, these changes did not produce detectable differences in Pb, Ni, or As removal. Thus, although homogenization modified mucilage dispersion and surface charge, all three samples provided sufficient interaction or flocculation capacity to reach the apparent removal plateau under the evaluated conditions.

The high As removal requires a broader interpretation than simple electrostatic attraction. Depending on its oxidation state and aqueous environment, As may occur as an anionic species and would not be expected to be strongly attracted to negatively charged mucilage. Nevertheless, Fox et al. [18] demonstrated that carboxyl, carbonyl, and hydroxyl groups of cactus mucilage participate in interactions with arsenate. In the present system, As removal may therefore have involved interactions with mucilage functional groups, association with cationic or inorganic domains, adsorption onto suspended phases, or incorporation of As-bearing particles into the flocs. The available data do not distinguish among these possibilities or establish direct binding to a specific arsenic species.

Cd exhibited a distinct response, with removal efficiencies ranging from approximately 40% to 94% and substantially greater variability than Pb, Ni, and As. Mucilage type had a significant effect, whereas dose and the mucilage type × dose interaction were not significant. Tukey′s test showed that CC had a higher marginal Cd removal than CB, while CA was statistically intermediate and did not differ significantly from either mucilage. The variability should first be considered in relation to the low initial Cd concentration and the proximity of some residual values to the quantification limit, because small absolute differences under these conditions produce comparatively large changes in calculated removal percentages [19].

Cd retention may have also been more sensitive to competition for accessible carboxylate domains, its distribution in the aqueous matrix, and the organization of the resulting flocs. Ibarra-Rodríguez et al. [38] likewise reported Cd as the least efficiently removed element by purified nopal pectin, although their experiments involved substantially higher concentrations, purified pectin, synthetic solutions, and different pH conditions. The comparison therefore indicates only that Cd can display a less favorable or more variable response than other elements; it does not demonstrate an identical mechanism.

The higher marginal Cd removal obtained with CC than with CB may reflect differences in the transient organization or floc-forming behavior of the reconstituted mucilages. CB and CC had the same average particle diameter, but differed in ζ potential and rheological response. These characteristics could influence polymer–particle contacts or incorporation into flocs, although the rheological measurements provide only indirect structural evidence because they were performed at a higher mucilage concentration and outside the jar-test conditions. Natural compositional variability, including the higher protein and ash contents measured in CC, may also have contributed. Consequently, the Cd response cannot be attributed exclusively to homogenization, and the results do not establish a progressive improvement with homogenization time or universal superiority of CC at each dose.

Zn showed the lowest percentage removal among the evaluated elements. Most treatments removed approximately 10–23%, whereas CA800 exhibited a higher mean accompanied by substantial variability. The two-way ANOVA detected a significant effect of dose, but no significant effect of mucilage type or mucilage type × dose interaction. Tukey′s test identified a difference between the marginal means at 400 and 800 mg·L^−1^, while 200 mg·L^−1^ showed an intermediate response and did not differ significantly from either dose. Because the response was non-monotonic, this result should not be interpreted as a proportional improvement in Zn removal with increasing mucilage concentration.

The comparatively low Zn removal percentage should be considered in relation to its much higher initial concentration. A removal of approximately 20% represented nearly 4.7 mg of Zn removed per liter, illustrating that percentage removal alone does not describe the absolute mass transferred from the liquid phase. Zinc is used in the evaluated beneficiation plant during upstream precious-metal recovery, an operational practice consistent with zinc cementation in Merrill–Crowe circuits [40,41,42]. This use may contribute to the comparatively high Zn concentration measured in the barren solution, although the magnitude of that contribution was not quantified. The higher Zn loading may consequently have limited the fraction of total Zn removable through the interaction and flocculation pathways available under the tested conditions.

The aqueous form of Zn may also have influenced its accessibility to the mucilage. Song et al. [43] emphasized that real cyanidation process streams contain more complex mixtures of dissolved species than the simulated solutions commonly used in laboratory studies. In their actual cyanide-leached gold wastewater, Zn was identified among the metal–cyanide complex species present in the solution. Although Zn speciation was not determined in the present barren solution, this evidence indicates that Zn in cyanidation streams cannot be assumed to occur entirely as free Zn^2+^. This distinction may partly explain the contrast between the limited Zn removal observed here and the high Zn^2+^ removal reported for purified nopal pectin in synthetic wastewater [38]. Different dissolved complexes, hydrolyzed species, and mineral- or colloid-associated fractions would be expected to differ in their interaction with the mucilage and susceptibility to incorporation into flocs.

The non-linear Zn response may additionally reflect the balance between the availability of polymeric retention domains and the efficiency of interparticle bridging. Increasing mucilage concentration provides additional functional groups and polymer chains, but may also alter particle-surface coverage and reduce the proportion of chain segments available to connect different particles. Under some conditions, extensive polymer coverage can favor stabilization rather than further floc growth [39]. Nevertheless, this interpretation remains tentative because the dose effect was strongly influenced by the highly variable CA800 result. The data neither establish 800 mg·L^−1^ as an optimal dose nor demonstrate that increasing mucilage concentration consistently improves Zn removal.

The combined removal pattern is consistent with interactions between accessible element species and deprotonated uronic acid domains, together with the capture of mineral-associated or colloidal fractions during floc formation and sedimentation. However, because the aqueous species and individual removal pathways were not quantified separately, this interpretation should be regarded as a mechanistic framework rather than a fully resolved mechanism.

### 2.7. FTIR Analysis of the Evaporated Barren Solution

**Figure 7:**
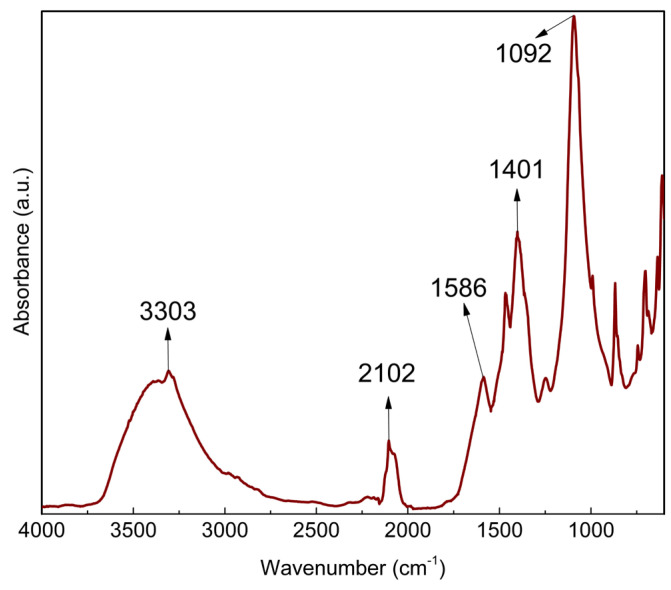
ATR-FTIR spectrum of the evaporated BS residue collected from a gold cyanidation plant. The spectrum provides a qualitative fingerprint of the dried saline process matrix and was used as a reference for comparison with the recovered flocs.

The ATR-FTIR spectrum of the evaporated BS provided a qualitative fingerprint of the nonvolatile inorganic matrix in which the mucilage treatment was performed (Figure 7). Because direct FTIR analysis of aqueous cyanidation streams is severely hindered by the intense infrared absorption of liquid water, evaporation was necessary to obtain spectral information. It is important to recognize, however, that evaporation alters the physical state of the sample and may modify the hydration, coordination, or distribution of dissolved components; therefore, the spectrum represents the dried residue rather than the original aqueous speciation of the BS.

The initial pH of the BS was 11.28, consistent with the highly alkaline conditions typically maintained in cyanidation and Merrill–Crowe circuits to prevent cyanide volatilization and optimize precious-metal recovery [42,43]. The spectrum exhibited several overlapping bands characteristic of a hydrated and chemically heterogeneous saline residue. The broad band centered at 3303 cm^−1^ and the signal near 1586 cm^−1^ are compatible with O–H stretching and H–O–H bending vibrations, respectively, indicating that associated water remained in the solid after evaporation. This behavior is consistent with the presence of hygroscopic inorganic salts and suggests that the dried process matrix retains hydrated components rather than forming an entirely anhydrous residue.

The feature near 1401 cm^−1^ is compatible with contributions from carbonate-containing phases, whose presence is chemically plausible given that strongly alkaline solutions readily absorb atmospheric CO_2_ during handling and evaporation. FTIR bands near 1421 and 876 cm^−1^ have been attributed to carbonate minerals in alkaline mineral matrices [44], and calcium carbonate and crystalline calcite have also been reported among the mineral components of *Opuntioideae* materials [2], although the origin and composition of those plant-derived phases differ from those of the present BS residue. The intense band near 1092 cm^−1^ is compatible with sulfate-containing salts or other inorganic oxyanions. Because several inorganic vibrations may overlap in this region, the exact contributors cannot be resolved from FTIR alone, and both assignments remain tentative.

Beyond mere functional-group identification, these spectral features provide relevant information about the physicochemical environment in which the treatment was conducted. At pH 11.28, the uronic acid carboxyl groups of the mucilage are predominantly deprotonated (COO^−^) [38], which is essential for electrostatic interactions with cationic species. However, the high concentration of carbonate and sulfate anions generates an environment of high ionic strength that can screen these charges and compete for cation binding. Conversely, the presence of calcium ions (derived from lime used in the cyanidation process) can act as ionic bridges between negatively charged polysaccharide chains, promoting polymer–particle aggregation and floc formation even in the presence of competing anions [39]. The spectral features are therefore consistent with a chemically complex matrix where ionic screening, competitive binding, and calcium-mediated bridging operate simultaneously.

A weak but reproducible band was observed near 2102 cm^−1^ in the raw duplicate spectra of the evaporated BS. This signal lies in a region commonly associated with C≡N stretching vibrations. Song et al. [43] reported bands near 2050–2053 cm^−1^ in an organic phase loaded with metal–cyanide complexes extracted from real cyanide-leached gold wastewater, although in that study the identities of the complexes were additionally supported by electrospray ionization mass spectrometry. Their findings support the spectral plausibility of a C≡N-containing contribution but do not establish the molecular identity of the band detected in the present BS residue. In alkaline cyanidation solutions, cyanide may exist as a free ion (CN^−^), as stable metal complexes (ferrocyanide, ferricyanide), or as complexes with base metals such as Zn, Cu, and Ni [42]. Since the mucilage is an anionic polyelectrolyte, greater affinity would be expected for cationic species or neutral complexes than for anions such as free CN^−^; however, physical capture of particles containing metal–cyanide complexes is also plausible. In the absence of complementary cyanide determination (e.g., volumetric titration or ion chromatography) or molecular-speciation analysis (such as electrospray ionization mass spectrometry), the band at 2102 cm^−1^ cannot be assigned conclusively to free cyanide or to a specific metal–cyanide complex. It is therefore described here as an unresolved feature compatible with a C≡N-containing contribution.

The reproducibility of the 2102 cm^−1^ signal confirms that it is a genuine feature of the dried BS residue. Its value in the present study lies in providing a qualitative comparison marker for the signals observed near 2104–2105 cm^−1^ in some recovered flocs (Section 2.8). It is important to note that, because the FTIR spectra were normalized to facilitate visual comparison (as described in Section 4.4), differences in the relative intensity of this band between samples should not be interpreted as quantitative differences in the concentration of the corresponding component, but rather as variations in its relative spectral contribution. Similarity between these spectral regions may indicate that one or more BS-derived components were incorporated into the solid matrix formed during coagulation–flocculation, but it does not independently demonstrate cyanide removal or identify the incorporated species. The FTIR fingerprint of the BS residue serves as a useful baseline that, together with the elemental and microstructural evidence presented in Section 2.8 and Section 2.9, supports the interpretation that the recovered flocs contain mucilage-derived organic material and inorganic components originating from the process stream.

### 2.8. FTIR Analysis of the Recovered Flocs

**Figure 8:**
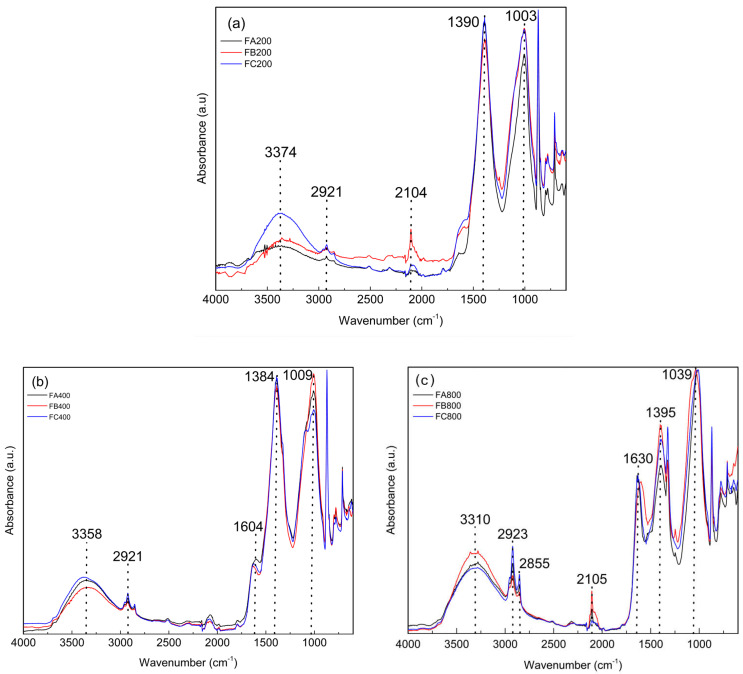
ATR-FTIR spectra of dried flocs recovered after jar tests using reconstituted nopal mucilage at final concentrations of (**a**) 200 mg·L^−1^, (**b**) 400 mg·L^−1^, and (**c**) 800 mg·L^−1^. FA, FB, and FC correspond to flocs obtained using CA, CB, and CC, respectively. CA is the non-homogenized control, whereas CB and CC correspond to mucilage homogenized for 30 and 60 s.

Figure 8 shows the ATR-FTIR spectra of the dried flocs recovered after coagulation–flocculation treatment of the BS. The flocs were designated according to the mucilage sample and dose used during treatment. Thus, FA200, FA400, and FA800 correspond to the flocs obtained using CA at 200, 400, and 800 mg·L^−1^, respectively. The same nomenclature was applied to the flocs obtained with CB and CC, designated as FB and FC. Each curve represents the mean of two independently acquired and normalized spectra.

Comparison of the spectra in Figure 8 with those of the original mucilage powders (Figure 4) and the evaporated BS residue (Figure 7) indicates that the recovered solids contained contributions from both materials. The simultaneous presence of characteristic polysaccharide bands and features absent from the original mucilage is consistent with the formation of a mixed organic–inorganic matrix during coagulation–flocculation and sedimentation.

Mucilage-related signals remained evident in the recovered flocs. These included the broad band at approximately 3310–3374 cm^−1^, associated with O–H stretching and hydrogen-bonded hydroxyl groups, and the signals near 2921–2923 and 2855 cm^−1^, attributed to aliphatic C–H stretching. The intense region between approximately 1003 and 1039 cm^−1^ is compatible with C–O and C–O–C vibrations of the polysaccharide backbone, although contributions from inorganic oxyanions originating from the BS may overlap in this region. These assignments agree with previous spectroscopic characterizations of Opuntia ficus-indica mucilage [32,33]. Their persistence supports the incorporation of mucilage-derived polysaccharide material into the recovered solids but does not establish preservation of the original macromolecular structure or molar mass of the polymer chains.

Variations were also observed in the regions near 1604–1630 and 1384–1395 cm^−1^. The former may include contributions from asymmetric stretching of carboxylate groups and bending vibrations of associated water, whereas the latter may contain contributions from symmetric carboxylate stretching and carbonate-containing phases [32,33,38]. Because both the mucilage powders and the evaporated BS residue exhibited signals in these regions, the corresponding bands in the recovered flocs cannot be assigned uniquely to either component. Their positions and relative spectral contributions are nevertheless consistent with variations in the organic, hydrated, and inorganic environments within the floc matrix.

At the alkaline pH of the BS, the uronic acid carboxyl groups of the mucilage would be expected to occur predominantly as COO^−^ [38]. These groups may interact with accessible positively charged species, while the polysaccharide chains may promote interparticle bridging and the capture of mineral-associated, precipitated, or colloidal material. Hydrogen bonding and physical entrapment within the developing floc network may also contribute to the formation of the recovered solids [38,39]. The FTIR changes are compatible with this combined interaction framework, although they do not distinguish among direct coordination, electrostatic association, adsorption onto inorganic phases, coprecipitation, and physical entrapment.

A weak feature near 2104–2105 cm^−1^ was evident in some recovered-floc spectra and was absent from the original mucilage powders. Its presence or absence was consistent between the corresponding individual duplicate spectra. Because no spectral smoothing was applied, the narrow feature cannot be attributed to a smoothing artifact. Its position is close to that of the unresolved band near 2102 cm^−1^ observed in the evaporated BS residue (Section 2.7) and lies within a region commonly associated with C≡N stretching vibrations. Song et al. [43] reported bands near 2050–2053 cm^−1^ for metal–cyanide complexes extracted from real cyanide-leached gold wastewater, although in that study the identities of the complexes were additionally supported by electrospray ionization mass spectrometry. Their findings support the spectral plausibility of a C≡N-containing contribution in this region but do not establish the molecular identity of the feature detected in the present flocs. In the absence of complementary cyanide determination or molecular-speciation analysis, it is more appropriately described as an unresolved feature also observed in the evaporated BS residue.

The 2104–2105 cm^−1^ feature displayed a reproducible but non-monotonic distribution among the treatments. At 200 mg·L^−1^, it was most clearly evident in FB200 and was weaker in FA200 and FC200. No distinct band was observed in this region at 400 mg·L^−1^, whereas a narrow feature was again evident at 800 mg·L^−1^. Its relative spectral contribution was therefore not directly proportional to mucilage dose or mucilage sample. This pattern may reflect treatment-dependent differences in the incorporation, distribution, or local chemical environment of one or more BS-derived components. Changes in mucilage dose may modify polymer coverage, interparticle bridging, aggregation, floc architecture, and physical entrapment, thereby producing a non-linear distribution of minor process-stream components within the recovered solids [38,39]. Differences among CA-, CB-, and CC-derived flocs may also arise from variations in mucilage particle size, surface charge, composition, and viscoelastic behavior (Section 2.1 and Section 2.2), which can influence the assembly of the organic–inorganic matrix.

The absence of a resolved band at 400 mg·L^−1^ does not necessarily indicate complete absence of the contributing component. A lower relative spectral contribution, redistribution within the floc matrix, changes in coordination or hydration, association with different inorganic phases, or band broadening may reduce its spectral definition. Likewise, the feature observed at 800 mg·L^−1^ may reflect a greater relative spectral contribution or a different local chemical environment rather than a simple increase in retained material.

It is important to note that because the FTIR spectra were normalized to facilitate visual comparison (as described in Section 4.4), differences in band intensity between samples cannot be converted directly into absolute concentrations, retained masses, or removal efficiencies. Nevertheless, the reproducibility of the unsmoothed duplicate spectra shows that the non-monotonic pattern was consistently observed under the evaluated treatment conditions and was not introduced by spectral smoothing. The correspondence between the unresolved features near 2102 cm^−1^ in the evaporated BS and 2104–2105 cm^−1^ in some recovered flocs provides qualitative evidence that one or more BS-derived components were incorporated into the recovered solid matrix. FTIR alone, however, does not demonstrate cyanide removal, conclusively identify the molecular species responsible for the band, or resolve its specific incorporation pathway. The FTIR evidence is best interpreted in conjunction with the elemental and microstructural analyses presented in Section 2.9, which provide complementary evidence for the incorporation of PTE-bearing inorganic domains within the floc matrix.

### 2.9. Microstructural and Elemental Analysis of the Recovered Flocs by ESEM-EDS

**Figure 9:**
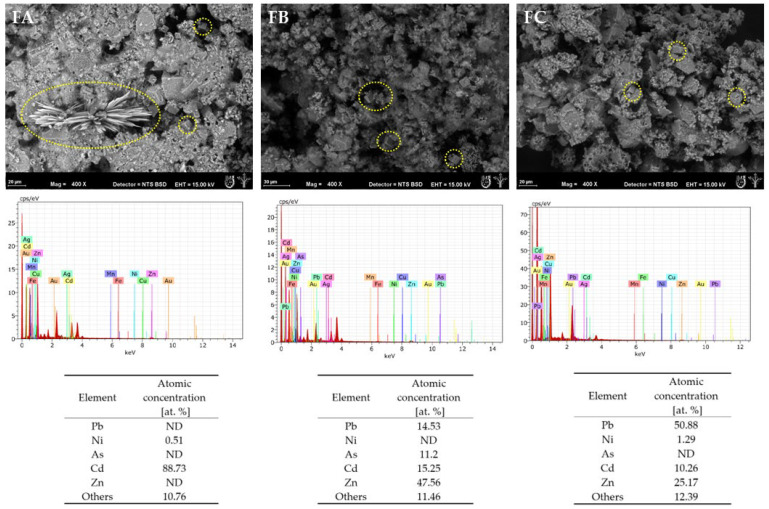
ESEM–EDS analysis of flocs recovered after treatment with 200 mg·L^−1^ of reconstituted nopal mucilage. FA200, FB200, and FC200 correspond to flocs obtained using CA, CB, and CC, respectively. The upper panels show representative backscattered-electron micrographs. Yellow dashed outlines indicate faceted, polyhedral, or acicular high-backscatter domains with crystal-like morphology selected for localized EDS analysis. The middle panels show the corresponding EDS spectra, and the lower tables report the localized semiquantitative atomic composition of the analyzed microregions. The reported atomic percentages do not represent the bulk composition of the complete flocs. ND: not detected in the selected microregion.

Figure 9 shows representative backscattered-electron micrographs and localized EDS analyses of the flocs recovered using 200 mg·L^−1^ of reconstituted nopal mucilage. This concentration was selected for presentation in the main text because it was the lowest dose evaluated and was already sufficient to reach the apparent removal plateau observed for Pb, Ni, and As (Section 2.6). The corresponding ESEM–EDS results for flocs obtained at 400 and 800 mg·L^−1^ are provided in the Supplementary Materials (Figures S1–S6).

The recovered solids exhibited heterogeneous structures composed of irregular aggregates, porous regions, and embedded domains with well-defined geometries. FA200 showed a comparatively compact matrix containing faceted particles and a prominent elongated acicular or rosette-like structure. FB200 displayed a more open and finely aggregated morphology, with small polyhedral domains distributed throughout the matrix, whereas FC200 contained coarser aggregates and several faceted, cubic, or polyhedral particles. These well-defined structures were not observed in the original spray-dried mucilage powders (Figure 6) and appeared only after treatment of the BS. Their morphology and relatively high backscattered-electron contrast are consistent with inorganic domains exhibiting crystal-like habits within the recovered flocs. However, their crystallinity and mineral-phase identity cannot be confirmed without complementary phase-identification techniques such as X-ray diffraction.

Localized EDS analysis detected the evaluated PTEs within the selected domains. The FA200 microregion showed a dominant Cd contribution of 88.73 at.% and a minor Ni contribution of 0.51 at.%. The analyzed region in FB200 contained Zn, Cd, Pb, and As at relative atomic contributions of 47.56, 15.25, 14.53, and 11.20 at.%, respectively. In FC200, the selected domain contained predominantly Pb and Zn at 50.88 and 25.17 at.%, together with Cd at 10.26 at.% and Ni at 1.29 at.%. The coexistence of several PTEs in FB200 and FC200 indicates that the flocs incorporated multicomponent inorganic material rather than forming compositionally uniform single-element solids.

The relatively high localized contribution of Zn should be interpreted together with its reference concentration and absolute mass removed. Although Zn showed the lowest percentage removal, approximately 19–21% at 200 mg·L^−1^ of mucilage (Section 2.6), its initial concentration in the BS was 23.46 mg·L^−1^. This represented approximately 4.47–4.91 mg Zn removed per liter, exceeding the corresponding amounts of Pb (approximately 1.96–1.98 mg·L^−1^) and Cd (approximately 0.43–0.47 mg·L^−1^). Therefore, a lower percentage removal does not necessarily imply a lower absolute amount transferred to the solid phase. The comparatively high Zn contribution detected in FB200 and FC200 is thus compatible with the greater Zn loading initially present in the BS and the amount available for incorporation into locally enriched domains.

The EDS atomic percentages represent relative elemental concentrations within the selected microregions and not the average or total composition of each floc. They cannot be converted directly into retained mass or used to rank the global removal performance of FA200, FB200, and FC200. Nevertheless, the detection of Pb, Ni, As, Cd, and Zn in the recovered solids corroborates the liquid-phase results obtained by atomic absorption spectrometry (Section 2.6). Taken together, the decrease in residual concentrations and the presence of PTE-bearing domains in the flocs confirm that the material containing these elements was transferred from the BS to the recovered solid phase.

Previous studies provide useful context for this behavior. Ibarra-Rodríguez et al. [38] observed metal-dependent morphologies in solids formed with purified nopal pectin, including microspherical structures associated with Pb, Cu, Cd, and Ca. Ni and Cr were detected at the surface without producing the same morphology, whereas the absence of surface-detected Zn was interpreted as possible encapsulation within the metal–pectin solids. Those authors also emphasized that SEM–EDS provides localized surface information and may not reproduce the global elemental composition measured by bulk analytical techniques. In the present study, the industrial BS contained multiple dissolved, complexed, mineral-associated, and colloidal components (Section 2.7); consequently, the observed morphologies cannot be assigned to individual elements solely from their shape.

The occurrence of faceted and acicular inorganic domains within an irregular mucilage-derived matrix, together with the multielemental composition of several analyzed regions, suggests that removal involved a substantial physical component. The evidence is compatible with the capture of precipitated, mineral-associated, or colloidal material through polymer adsorption, interparticle bridging, aggregation, physical entrapment, and subsequent sedimentation. López-Maldonado et al. [39] similarly described coagulation–flocculation in complex wastewaters as a process governed by the combined effects of surface charge, polyelectrolyte dose, chemical affinity, and physical particle destabilization and aggregation. Parga et al. [41] used SEM–EDS to demonstrate the association of recovered metals with inorganic solids generated during treatment of a cyanide-barren solution. Although their electrocoagulation process involved iron-rich phases and differs from the mucilage-based treatment used here, their results illustrate how PTE-containing species in cyanidation streams can become concentrated on or within secondary solids. Duan et al. [44] likewise showed that newly formed calcium silicate hydrate and carbonate phases could fill pores and immobilize metals in treated cyanide tailings, although phase identity in that study was supported by XRD and other complementary techniques.

The ESEM–EDS results are consistent with the ATR-FTIR evidence (Section 2.8) of a mixed organic–inorganic floc matrix containing mucilage-derived polysaccharide material and components originating from the BS. The combination of spectroscopic, elemental, and microstructural evidence supports a removal mechanism dominated by aggregation, polymer bridging, incorporation of PTE-containing inorganic particles, physical entrapment, and sedimentation. Direct coordination or electrostatic association between accessible element species and deprotonated carboxylate groups (Section 2.7) may also have contributed, but the present data do not allow their individual contributions to be separated. The complementary nature of the FTIR and ESEM-EDS evidence strengthens the overall interpretation that the recovered flocs represent a complex organic–inorganic assembly formed through the interaction of the mucilage with the chemically diverse components of the BS.

## 3. Conclusions

Mechanical homogenization of the aqueous nopal mucilage extract before spray drying modified the physicochemical, colloidal, microstructural, and rheological properties of the resulting powders and their water-reconstituted dispersions. Homogenization for 30 and 60 s reduced water activity and average hydrodynamic diameter and altered the ζ potential, while the characteristic polysaccharide-related FTIR profile and overall thermal behavior were retained. The effects were not proportional to processing time, as the 30 s treatment exhibited the most negative ζ potential and enhanced dispersion, whereas the 60 s treatment showed distinct viscoelastic behavior consistent with a different supramolecular organization. The combined colloidal and rheological results support the interpretation that short-term mechanical shear primarily promotes dispersion, while prolonged exposure may induce weak physical associations among polysaccharide chains.

At 10% (*w·v*^−1^), the reconstituted mucilages exhibited frequency-dependent viscoelastic behavior, generally shifting from viscous predominance at lower frequencies toward elastic predominance at higher frequencies. This response is consistent with a transient gel-like organization sustained by physical entanglements, hydrogen bonding, and hydrated polysaccharide domains, rather than with a strong and permanently crosslinked gel. Homogenization modified this gel-like behavior and the relaxation characteristics of the mucilage. Because the rheological measurements were performed at a substantially higher concentration than that used in the jar tests, the existence of an equivalent gel-like network under the treatment conditions cannot be assumed directly.

All three reconstituted mucilages removed more than 98% of the PTEs Pb, Ni, and As from the real cyanidation barren solution at all evaluated doses. The lowest dose, 200 mg·L^−1^, was already sufficient to reach the apparent removal plateau for these PTEs. Cadmium showed a more variable response and a significant effect of mucilage type, whereas zinc exhibited lower percentage removal and a significant but non-monotonic dose effect. Nevertheless, because of its substantially higher reference concentration, approximately 20% zinc removal represented nearly 4.7 mg·L^−1^ transferred from the liquid phase, exceeding the absolute amounts removed for lead and cadmium.

The decrease in residual PTE concentrations, together with the localized detection of Pb, Ni, As, Cd, and Zn in the recovered flocs by ESEM–EDS, corroborates the transfer of PTE-bearing material from the barren solution to the solid phase. The flocs contained heterogeneous mucilage-derived matrices with embedded faceted, polyhedral, cubic, and acicular inorganic domains exhibiting crystal-like habits. Their localized and frequently multielemental composition is consistent with an important contribution from physical processes, including aggregation, polymer bridging, incorporation of precipitated or mineral-associated particles, physical entrapment, and sedimentation. Interactions between accessible PTE species and deprotonated mucilage carboxylate groups may also have contributed, although the relative importance of the individual pathways could not be resolved.

ATR-FTIR further supported the formation of mixed organic–inorganic flocs containing mucilage-derived polysaccharide material and components originating from the barren solution. The reproducible feature near 2102–2105 cm^−1^ provided a qualitative spectral link between the evaporated barren solution residue and some recovered flocs. However, this unresolved feature can only be described as compatible with a C≡N-containing contribution and does not independently demonstrate cyanide removal or identify a specific cyanide-containing species. Complementary cyanide-speciation analysis would be required to establish the fate of cyanide species during treatment.

The combined spectroscopic, elemental, and microstructural evidence supports a removal mechanism dominated by aggregation, polymer bridging, incorporation of inorganic particles, physical entrapment, and sedimentation. The high ionic strength and presence of competing anions and calcium ions in the barren solution matrix likely modulate these interactions, explaining why the colloidal differences among the mucilage treatments did not translate into universal improvements in PTE removal.

Overall, this proof of concept demonstrates the potential of spray-dried and water-reconstituted nopal mucilage as a natural hydrocolloid with coagulant–flocculant functionality and the capacity to develop transient gel-like viscoelastic behavior under concentrated reconstitution. Mechanical homogenization with a household blender provided a simple and accessible means of modifying its colloidal, supramolecular, and gel-related properties, although these changes did not result in a universal improvement in PTE removal across all elements evaluated. The non-linear effects of homogenization time on mucilage properties underscore the importance of processing standardization for the reproducible production of functional biopolymer-based coagulants.

Further studies should compare the mucilage with conventional coagulants and examine the influence of operating conditions, storage stability, regeneration, floc management, and scale-up under industrially relevant conditions. Comprehensive cyanide-speciation analysis and solid-phase spectroscopic techniques would be valuable in resolving the specific binding mechanisms and the fate of cyanide species. The present results provide a basis for further evaluating mechanically processed nopal mucilage as a multifunctional hydrocolloid for the treatment of chemically complex cyanidation process streams.

## 4. Materials and Methods

### 4.1. Mucilage Extraction and Pretreatment

*O. ficus-indica* cladodes with approximately 15 days of growth were collected from San Juan Tlacotenco, Tepoztlán, Morelos, Mexico (19°01′05.2″ N, 99°05′43.6″ W). The cladodes were stored at 4 °C until processing. After removing spines, the cladodes were washed with distilled water and cut into pieces (3.5 × 2.5 × 0.5 cm). The pieces were hydrated with distilled water in a 1:1 (*w·v*^−1^) ratio for 4 h at room temperature (35 ± 2 °C) protected from light. The mixture was filtered through a stainless-steel mesh (No. 60), and the liquid mucilage extract was recovered.

The mucilage extract was processed in 400 mL batches. Mechanical homogenization was performed using a household blender (Oster, 250 W) placed at the bottom center of the vessel, applying a nominal power density of 625 W·L^−1^. Homogenization was carried out for 0 s (CA, non-homogenized control), 30 s (CB, homogenized for 30 s), and 60 s (CC, homogenized for 60 s). The temperature of the extract was monitored to ensure it did not exceed 30 °C during processing. All samples were then spray-dried using a pilot-scale atomizer (Niro) with a 13 cm diameter disk operated at 23,000 rpm. The inlet air temperature was set at 230 ± 5 °C, and the outlet temperature was maintained at 75 ± 5 °C by controlling the feed rate with a peristaltic pump (Masterflex L/S 7510-00, Cole-Parmer, Vernon Hills, IL, USA). The resulting powders were stored in sealed polyethylene bags in a desiccator with silica gel.

### 4.2. Physicochemical Characterization

#### 4.2.1. Proximate Composition

Moisture, protein (Kjeldahl method, N × 6.25), ash, and lipid contents were determined according to AACC methods 44-19.01, 46-13.04, and 44-19.01, respectively [45]. Carbohydrate content was calculated by difference.

#### 4.2.2. Water Activity

Water activity was measured at ambient temperature (36 ± 1 °C) using a WA 60 A hygrometer (AMTAST, Guangzhou, China) following the method described by Du Toit et al. [46].

#### 4.2.3. Hydrodynamic Diameter and Zeta Potential

Mucilage powders were reconstituted at 0.13% (*w·v*^−1^) in HPLC-grade water at 25 °C and stirred for 1 h to ensure complete solubilization. The average hydrodynamic diameter and zeta potential (ζ) were determined by dynamic light scattering (DLS) using a Zetasizer Nano ZS (Malvern Instruments, Worcestershire, UK) at 25 °C [22]. The natural pH of the reconstituted dispersion was 6.8 ± 0.2, and no pH adjustment was made.

### 4.3. Rheological Measurements

Dispersions for rheology were prepared at 10% (*w·v*^−1^) by dissolving 5 g of powder in 50 mL of distilled water under gentle magnetic stirring for 30 min. This higher concentration was necessary to obtain measurable viscoelastic signals. All rheological measurements were performed at 20 °C.

#### Dynamic Oscillatory Measurement

Dynamic oscillatory measurements were performed using a rotational rheometer (Kinexus, Malvern Instruments Ltd., Malvern, Worcestershire, UK) equipped with a parallel-plate geometry (40 mm diameter) with a gap of 1 mm. The linear viscoelastic region (LVR) was determined by strain amplitude sweeps at 1 Hz at 20 °C. Frequency sweeps were conducted from 0.1 to 10 Hz at 0.5% strain within the LVR. The storage modulus (G′), loss modulus (G″), and complex viscosity (η*) were recorded. The complex viscosity was calculated according to Equation (1):

(1) $$\eta^{*}=\frac{\left|G^{*}\right|}{\omega }=\frac{\sqrt{G^{\prime 2}+G^{\prime \prime 2}}}{\omega }$$

where η* is the complex viscosity, |G*| is the complex modulus, and ω is the angular frequency expressed in rad s^−1^. The angular frequency was calculated as ω=2πf, where f is the oscillation frequency in Hz.

### 4.4. Fourier Transform Infrared Spectroscopy (FTIR)

ATR-FTIR spectra of the powdered mucilage samples were recorded using an IRAffinity-1 spectrometer (Shimadzu, Kyoto, Japan) equipped with a diamond attenuated total reflectance accessory. Samples were analyzed directly at room temperature without additional preparation. Spectra were acquired over the range of 4000–400 cm^−1^ using 100 scans at a spectral resolution of 4 cm^−1^ [1]. Two independent spectra were obtained for each sample. Each spectrum was individually normalized to the 0–1 range using OriginPro 9.0, and the mean of the two normalized spectra was used for graphical presentation.

### 4.5. Thermogravimetric Analysis (TGA)

Thermogravimetric analysis (TGA) was conducted using a Q5000 IR thermogravimetric analyzer (TA Instruments, New Castle, DE, USA) to assess the thermal stability of nopal mucilage powders. The samples (2–5 mg) were placed in platinum crucibles and heated from 25 °C to 650 °C at a heating rate of 10 °C·min^−1^ under a nitrogen atmosphere (flow rate 50 mL min^−1^). The maximum degradation temperature (T_max_) was determined from the peak of the first derivative (DTG) curve.

### 4.6. Microstructure and Digital Image Analysis of Mucilage Powders

#### 4.6.1. Scanning Electron Microscopy (SEM)

Mucilage powder samples were observed using an Inspect F50 scanning electron microscope (FEI, Hillsboro, OR, USA) operated at an accelerating voltage of 5 kV. Prior to observation, the samples were mounted on carbon adhesive tape and sputter-coated with a thin gold/palladium (Au/Pd) layer to reduce charging. Images were acquired using the Everhart–Thornley detector in secondary electron mode at 2000× magnification. All micrographs were stored in uncompressed TIFF format at a resolution of 2048 × 1887 pixels.

#### 4.6.2. Digital Image Analysis

SEM micrographs were processed using ImageJ version 1.54 g (National Institutes of Health, Bethesda, MD, USA) following the methodology described by Avila-Reyes et al. [34] and Moreno-León et al. [35]. For each treatment (CA, CB, CC), 160 regions of interest of 50 × 50 pixels were randomly selected from the original images, ensuring that each subimage contained only the particle surface without edges or background.

Texture parameters were extracted using the gray-level co-occurrence matrix (GLCM) algorithm. The following descriptors were calculated:

- Angular second moment (ASM), a measure of textural uniformity (Equation (2)):

(2) $$ASM=\sum_{i=0}^{G-1}\sum_{j=0}^{G-1}{\left\{P\left(i,j\right)\right\}}^{2}\;$$

- Contrast, which quantifies local variations in gray level values (Equation (3)):

(3) $$Constrast=\sum_{n=0}^{G-1}n^{2}\left\{\sum_{i=1}^{G}\sum_{j=1}^{G}P\left(i,j\right)\right\}\;,\;\left|i-j\right|=n\;$$

- Inverse difference moment (IDM), representing local homogeneity (Equation (4)):

(4) $$IDM=\sum_{i=0}^{G-1}\sum_{j=0}^{G-1}\frac{1}{1+{\left(i+j\right)}^{2}}P\left(i,j\right)\;$$

- Entropy, which measures the disorder or randomness of the image (Equation (5)):

(5) $$Entropy=-\sum_{i=0}^{G-1}\sum_{j=0}^{G-1}P\left(i,j\right)\cdot \log\left(P\left(i,j\right)\right)$$

where $P\left(i,j\right)$ is the probability of occurrence of a pair of gray levels i and j at given displacement distance and direction.

Fractal dimension (FD) was determined using the shifting differential box counting (SDBC) method with the FracLac plugin in ImageJ. The fractal dimension (FD) was calculated as an indicator of surface geometric complexity.

### 4.7. Coagulation–Flocculation Treatment of the Cyanidation Barren Solution

#### 4.7.1. Characterization of the BS

The BS was obtained from a gold-processing plant located in Pachuca, Hidalgo, Mexico, and stored at 4 °C until use.

The initial pH of the BS was measured before treatment using a PHS-3BW pH meter (BANTE Instruments Co., Ltd., Shanghai, China). The instrument was calibrated at three points using standard buffer solutions of pH 4.0, 7.0, and 10.0, according to the manufacturer′s instructions. The initial pH of the BS was 11.28, and no external pH adjustment was performed before or during the coagulation–flocculation assays.

An aliquot of the untreated BS was dried at 40 °C for 24 h. The resulting solid residue was analyzed by diamond-ATR-FTIR under the instrumental conditions described in Section 4.4.

#### 4.7.2. Coagulation–Flocculation Assays and PTE Removal

For each jar test, 450 mL of BS was combined with 50 mL of an aqueous mucilage dispersion to obtain a final volume of 500 mL and final mucilage concentrations of 200, 400, or 800 mg·L^−1^. A mucilage-free control was prepared by replacing the mucilage dispersion with 50 mL of HPLC-grade water. The control and mucilage-containing treatments therefore contained the same volume of BS, the same volume of aqueous medium, and the same final volume.

The assays were conducted using a programmable PB-700 jar-test apparatus (Phipps & Bird, Richmond, VA, USA). The mucilage-free control and mucilage-containing treatments were subjected to rapid mixing at 150 rpm for 1.5 min, followed by slow mixing at 25 rpm for 20 min and settling for 60 min [47]. After settling, a 30 mL aliquot of the supernatant was collected from each vessel for elemental analysis.

The concentrations of Pb, Ni, As, Cd, and Zn in the processed mucilage-free control and in the supernatants recovered after treatment were determined by flame atomic absorption spectrometry using an AAnalyst 100 spectrometer (PerkinElmer, Inc., Waltham, MA, USA) equipped with hollow-cathode lamps and an air–acetylene burner. Analytical wavelengths, lamp currents, and spectral slit widths were selected according to the manufacturer′s recommendations for each element. Quantification was performed according to NMX-AA-051-SCFI-2016 [48].

The removal efficiency for each PTE for each experimental replicate was calculated according to Equation (6):

(6) $$\%R_{i}=\frac{{\bar{C}}_{SB}-C_{f,i}}{{\bar{C}}_{SB}}\;x\;100\;$$

where ${\bar{C}}_{BS}$ is the mean concentration of the corresponding PTE measured in the processed mucilage-free control BS, and $C_{f,i}$ is the residual PTE concentration measured in replicate i after treatment. The mean removal efficiency for each experimental condition was calculated according to Equation (7):

(7) $$\bar{\%R}=\frac{1}{n}\sum_{i=1}^{n}\%R_{i}\;$$

where (n) is the number of experimental replicates.

The LOD and LOQ values were, respectively, 0.0110 and 0.0335 mg·L^−1^ for Pb, 0.0124 and 0.0376 mg·L^−1^ for Ni, 0.0998 and 0.302 mg·L^−1^ for As, 0.0183 and 0.0555 mg·L^−1^ for Cd, and 0.0229 and 0.0693 mg·L^−1^ for Zn. Concentrations between the corresponding LOD and LOQ were retained for the removal calculations and interpreted as semiquantitative estimates. Negative responses obtained after analytical blank correction were assigned a value of zero before calculating removal efficiency.

#### 4.7.3. FTIR Characterization of the Recovered Flocs

The recovered flocs were dried at 40 °C for 24 h and analyzed by FTIR under the instrumental conditions described in Section 4.4.

#### 4.7.4. Environmental Scanning Electron Microscopy and Energy-Dispersive X-Ray Spectroscopy of Recovered Flocs

Floc samples obtained after treatment with reconstituted nopal mucilage at doses of 200, 400, and 800 mg·L^−1^ were mounted on aluminum stubs using double-sided conductive carbon tape and examined without conductive coating using an environmental scanning electron microscope (ESEM; EVO LS10, Carl Zeiss Microscopy GmbH, Jena, Germany).

The analyses were performed at an accelerating voltage of 15 kV and a chamber pressure of 80 Pa using water vapor. Representative grayscale images were acquired at 400× magnification using a backscattered-electron detector (NTS BSD). Selected crystalline domains within the floc matrix were analyzed by energy-dispersive X-ray spectroscopy (EDS). The resulting spectra were used for elemental identification and determination of the localized semiquantitative atomic composition of the selected microregions.

### 4.8. Statistical Analysis

Quantitative determinations were performed in triplicate, unless otherwise stated, and results are expressed as mean ± standard deviation. Proximate composition, water activity, zeta potential, particle size, PDI, and microstructural parameters were analyzed by one-way ANOVA, followed by Tukey′s test when significant differences were detected (*p* < 0.05).

Rheological curves represent the mean of three independent frequency sweeps per treatment. Because successive frequency measurements were not independent, no point-by-point ANOVA was performed, and rheological behavior was interpreted descriptively.

Removal efficiencies for Pb, Ni, As, Cd, and Zn were analyzed separately by two-way ANOVA using mucilage sample, dose, and their interaction as fixed factors. Significant main effects were evaluated using Tukey′s test (*p* < 0.05). All analyses were performed using OriginPro 9.0.

## Figures and Tables

**Table 1:** Proximate composition and physicochemical properties of nopal mucilage obtained at different homogenization times.

Parameter	CA	CB	CC
Moisture * (%)	2.70 ± 0.03 ^a^	2.74 ± 0.08 ^a^	2.68 ± 0.17 ^a^
Protein * (%)	9.22 ± 0.20 ^b^	8.64 ± 0.20 ^b^	9.92 ± 0.40 ^a^
Ash * (%)	27.56 ± 0.37 ^a^	27.49 ± 0.32 ^a^	29.95 ± 0.55 ^b^
Lipids * (%)	3.93 ± 0.20 ^a^	4.10 ± 0.18 ^a^	4.15 ± 0.21 ^a^
Carbohydrates * (%)	56.59 ± 0.61	57.03 ± 0.43	53.30 ± 0.73
Water activity (a_w_)	0.135 ± 0.007 ^a^	0.110 ± 0.000 ^b^	0.115 ± 0.007 ^b^
Zeta potential (mV)	−15.7 ± 0.3 ^a^	−18.3 ± 0.4 ^b^	−17.0 ± 0.5 ^c^
Average particle diameter (µm)	1.8 ± 0.2 ^a^	1.3 ± 0.1 ^b^	1.3 ± 0.1 ^b^
Polydispersity index	0.60 ±0.17 ^a^	0.63 ± 0.04 ^a^	0.60 ± 0.10 ^a^

* Values are expressed as mean ± standard deviation. Proximate composition values are expressed as g per 100 g of sample. Carbohydrates were calculated by difference, as commonly reported for proximate composition analysis of *Opuntia ficus-indica* mucilage [10,27]. CA: control without homogenization; CB: sample homogenized for 30 s; CC: sample homogenized for 60 s. Different superscript letters within the same row indicate significant differences among samples according to Tukey′s test (*p* < 0.05). Water activity (a_w_) was measured at 36 ± 1 °C. Zeta potential and particle size were determined in reconstituted mucilage dispersions (0.13% *w·v*^−1^) at their natural pH (6.8 ± 0.2).

**Table 2:** Thermal parameters of nopal mucilage powders obtained at different homogenization times.

Sample	T5% (°C)	T10% (°C)	Tmax (°C)	DTGmax (%/°C)
CA	142.2	172.6	215.2	−0.2587
CB	136.1	171.3	218.4	−0.2455
CC	137.6	172.3	219.3	−0.2425

CA: control without homogenization; CB: sample homogenized for 30 s; CC: sample homogenized for 60 s. T5% and T10% correspond to the temperature at which 5% and 10% mass loss occurred, respectively. Tmax corresponds to the temperature of the maximum degradation rate obtained from DTG. DTGmax corresponds to the maximum mass loss rate.

**Table 3:** Effect of homogenization time on texture parameters and fractal dimension of nopal mucilage.

Sample	Contrast	Entropy	ASM (×10^−4^)	IDM	FD
CA	469.17 ± 136.16 ^a^	7.81 ± 0.20 ^a^	5.00 ± 1.28 ^a^	0.094 ± 0.015 ^a^	2.23 ± 0.06 ^a^
CB	375.56 ± 97.56 ^b^	7.45 ± 0.23 ^b^	7.47 ± 1.83 ^b^	0.134 ± 0.015 ^b^	2.16 ± 0.05 ^b^
CC	353.08 ± 95.01 ^b^	7.42 ± 0.25 ^b^	7.77 ± 2.32 ^b^	0.135 ± 0.017 ^b^	2.17 ± 0.06 ^b^

Data are expressed as mean ± standard deviation (*n* = 160). Different superscript letters within the same column indicate significant differences according to Tukey′s test (*p* < 0.05). ASM: angular second moment (uniformity); IDM: inverse difference moment (local homogeneity); FD: total fractal dimension.

**Table 4:** Removal efficiency and residual concentration of PTEs after treatment with reconstituted nopal mucilage.

Sample		CA			CB			CC		
Dose (mg/L)		200	400	800	200	400	800	200	400	800
Pb	Removal (%)	98.08 ± 1.40	98.23 ± 1.15	98.65 ± 0.14	98.46 ± 0.23	98.89 ± 0.29	99.08 ± 0.61	99.09 ± 0.61	98.59 ± 0.94	98.44 ± 0.73
	Residual (mg/L)	0.038 ± 0.028	0.036 ± 0.023	0.027 ± 0.003 †	0.031 ± 0.005 †	0.022 ± 0.006 †	0.018 ± 0.012 †	0.018 ± 0.012 †	0.028 ± 0.019 †	0.031 ± 0.015 †
Ni	Removal (%)	99.23 ± 0.92	99.21 ± 0.91	99.25 ± 0.79	98.97 ± 0.87	99.10 ± 1.16	99.20 ± 0.92	99.22 ± 0.84	98.99 ± 0.69	99.12 ± 0.80
	Residual (mg/L)	0.038 ± 0.046	0.040 ± 0.045	0.037 ± 0.04 †	0.051 ± 0.044	0.045 ± 0.058	0.040 ± 0.046	0.039 ± 0.042	0.050 ± 0.034	0.044 ± 0.04
As	Removal (%)	99.96 ± 0.08	98.90 ± 1.56	98.28 ± 2.01	99.81 ± 0.39	99.38 ± 1.24	98.83 ± 1.92	99.90 ± 0.20	100.00 ± 0.00	99.92 ± 0.16
	Residual (mg/L)	0.004 ± 0.008 ‡	0.110 ± 0.156 †	0.172 ± 0.201 †	0.02 ± 0.039 ‡	0.062 ± 0.124 ‡	0.117 ± 0.192 †	0.01 ± 0.02 ‡	<LOD ‡	0.008 ± 0.016 ‡
Cd	Removal (%)	86.31 ± 27.38 ^ab^	76.81 ± 26.97 ^ab^	82.66 ± 21.17 ^ab^	87.12 ± 25.76 ^b^	39.93 ± 25.81 ^b^	59.40 ± 21.98 ^b^	93.16 ± 13.67 ^a^	93.75 ± 12.50 ^a^	89.02 ± 21.96 ^a^
	Residual (mg/L)	0.069 ± 0.137	0.116 ± 0.135	0.087 ± 0.106	0.064 ± 0.129	0.300 ± 0.129	0.203 ± 0.11	0.034 ± 0.068†	0.031 ± 0.063†	0.055 ± 0.11†
Zn	Removal (%)	20.93 ± 5.16 ^ab^	18.60 ± 1.76 ^b^	49.80 ± 39.98 ^a^	20.26 ± 4.21 ^ab^	10.13 ± 2.53 ^b^	20.28 ± 1.91 ^a^	19.06 ± 3.13 ^ab^	10.93 ± 0.92 ^b^	22.50 ± 4.63 ^a^
	Residual (mg/L)	18.552 ± 1.210	19.099 ± 0.412	11.779 ± 9.381	18.710 ± 0.987	21.086 ± 0.594	18.706 ± 0.447	18.992 ± 0.734	20.899 ± 0.216	18.183 ± 1.086

CA: non-homogenized control; CB: mucilage homogenized for 30 s; CC: mucilage homogenized for 60 s. The numbers 200, 400, and 800 indicate the final concentration of reconstituted mucilage added to the barren solution (mg·L^−1^). Values are expressed as mean ± standard deviation (*n* = 3). Two-way ANOVA was performed separately for each element, with mucilage type and dose as fixed factors. No significant mucilage × dose interactions were detected. For Cd, different lowercase letters indicate significant differences among the marginal means of mucilage type, averaged across doses (CA, ab; CB, b; CC, a). For Zn, different lowercase letters indicate significant differences among the marginal means of dose, averaged across mucilage types (200 mg·L^−1^, ab; 400 mg·L^−1^, b; 800 mg·L^−1^, a), according to Tukey′s test (*p* < 0.05). No significant effects were detected for Pb, Ni, or As. † Mean residual concentration between the LOD and LOQ; ‡ mean residual concentration below the LOD. Values below the LOQ should be interpreted as semiquantitative estimates.

## Data Availability

Dataset available on request from the authors.

## Supplementary Materials

The following supporting information can be downloaded at: https://www.mdpi.com/article/10.3390/gels12070569/s1, Figure S1: ESEM micrograph and EDS analysis of flocs obtained with CA at 400 mg·L^−1^ (FA400); Figure S2: ESEM micrograph and EDS analysis of flocs obtained with CA at 800 mg·L^−1^ (FA800); Figure S3: ESEM micrograph and EDS analysis of flocs obtained with CB at 400 mg·L^−1^ (FB400); Figure S4: ESEM micrograph and EDS analysis of flocs obtained with CB at 800 mg·L^−1^ (FB800); Figure S5: ESEM micrograph and EDS analysis of flocs obtained with CC at 400 mg·L^−1^ (FC400); Figure S6: ESEM micrograph and EDS analysis of flocs obtained with CC at 800 mg·L^−1^ (FC800).

## Abbreviations

The following abbreviations are used in this manuscript:

CA	Non-homogenized control mucilage
CB	Mucilage homogenized for 30 s
CC	Mucilage homogenized for 60 s
ζ	Zeta potential
PTEs	Potentially toxic elements
BS	Barren solution
a_w_	Water activity
PDI	Polydispersity index
G″	Loss modulus
G′	Storage modulus
tan δ	Loss tangent
η*	Complex viscosity
G*	Complex modulus
ATR-FTIR	Attenuated total reflectance Fourier transform infrared spectroscopy
TGA	Thermogravimetric analysis
DTG	Derivative thermogravimetry
Tmax	Temperature of maximum degradation rate
DTGmax	Maximum mass loss rate
T5%	Temperature at 5% mass loss
T10%	Temperature at 10% mass loss
SEM	Scanning electron microscopy
GLCM	Gray level co-occurrence matrix
FD	Fractal dimension
ASM	Angular second moment
IDM	Inverse difference moment
SD	Standard deviation
LOD	Limit of detection
LOQ	Limit of quantification
FA	Flocs obtained using CA mucilage
FB	Flocs obtained using CB mucilage
FC	Flocs obtained using CC mucilage
AACC	American Association of Cereal Chemists
DLS	Dynamic light scattering
LVR	Linear viscoelastic region
SDBC	Shifting differential box counting
