# Supplementary material for: Effect of Mechanical Homogenization on Nopal Mucilage for the Treatment of a Real Cyanidation Barren Solution
Source: Gels. 2026 Jun 27;12(7):569. doi: 10.3390/gels12070569 (PMC13409068; doi:10.3390/gels12070569)
Supplement: Supplementary file 1 [file gels-12-00569-s001.zip › gels-4369055-supplementary.pdf]

Effect of Mechanical Homogenization on Nopal Mucilage for the Treatment of a Real Cyanidation Barren Solution

Allison Vianey Valle-Bravo [1], Brenda Hildeliza Camacho-Díaz [1], Denis Rodrigue [2,*], Glenda Pacheco-Vargas [1], Francisco Rodríguez-González [1], Isidra Guadalupe Ruiz-Martínez [1] and Javier Solorza-Feria [1,*]

FA400

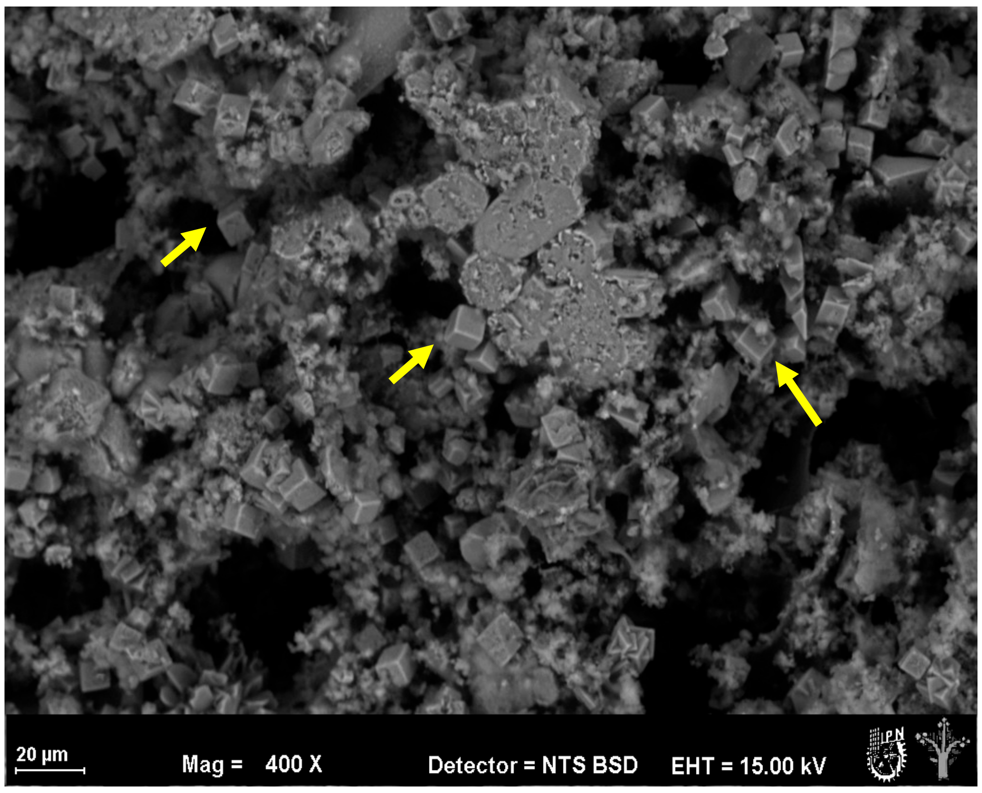

Spectrum: Acquisition 2906

Element	Atom. C [at.%]
Iron	3.96
Copper	4.63
Nickel	0.30
Manganese	0.76
Lead	13.42
Zinc	55.75
Cadmium	19.40
Silver	0.00
Gold	1.78
Total:	100.00

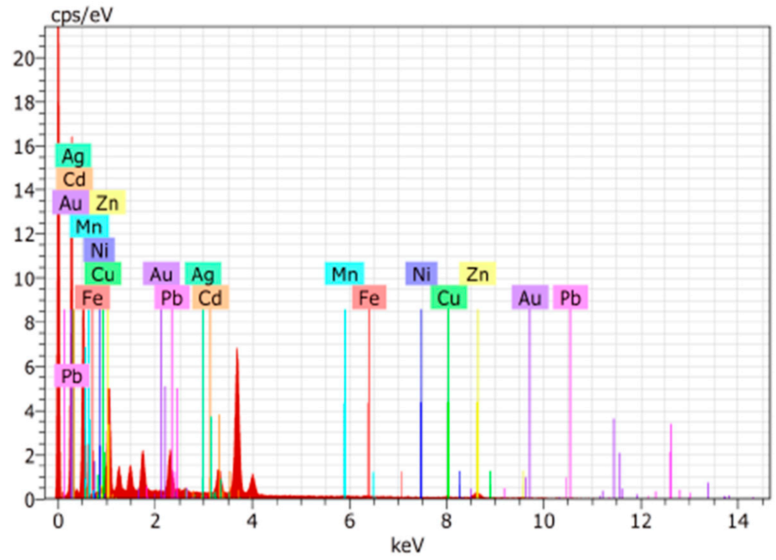

Figure S1. ESEM micrograph and EDS analysis of flocs obtained with CA at 400 mg·L^{-1} (FA400). Yellow arrows indicate selected inorganic domains.

FA800

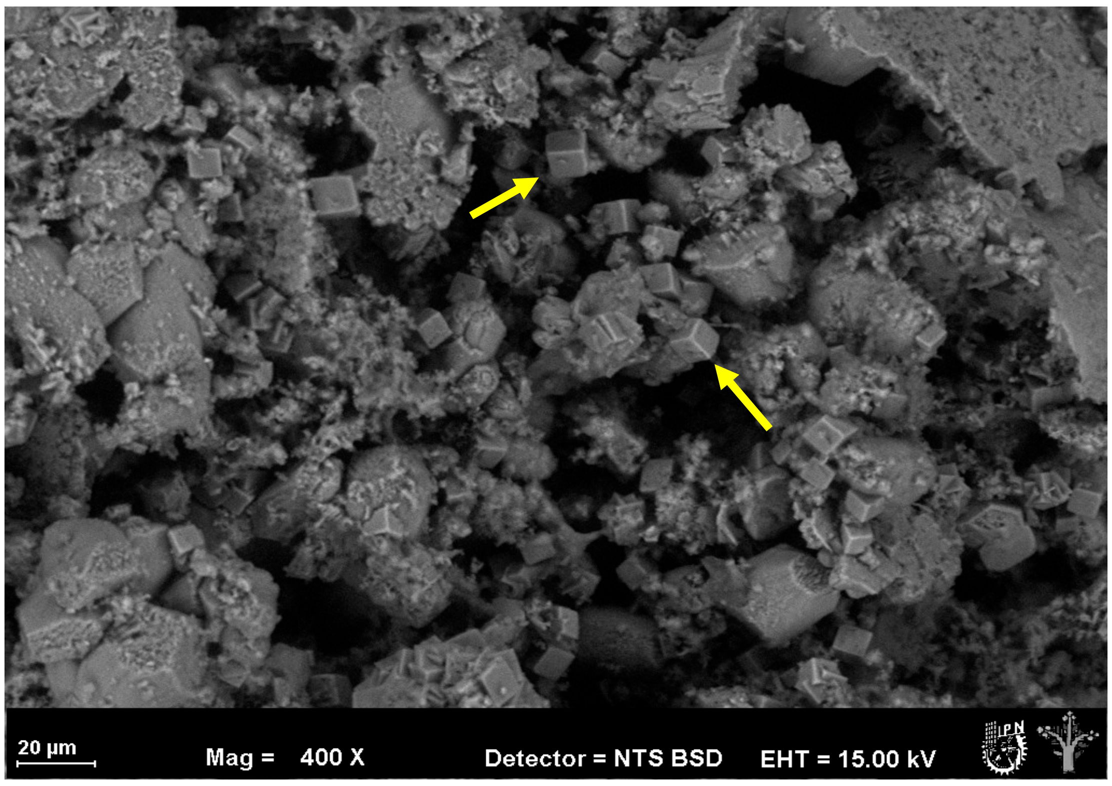

Spectrum: Acquisition 2908

Element	Atom. C [at.%]
Iron	2.67
Nickel	0.00
Copper	2.46
Silver	0.00
Gold	1.47
Zinc	69.55
Cadmium	11.01
Lead	12.84
Total:	100.00

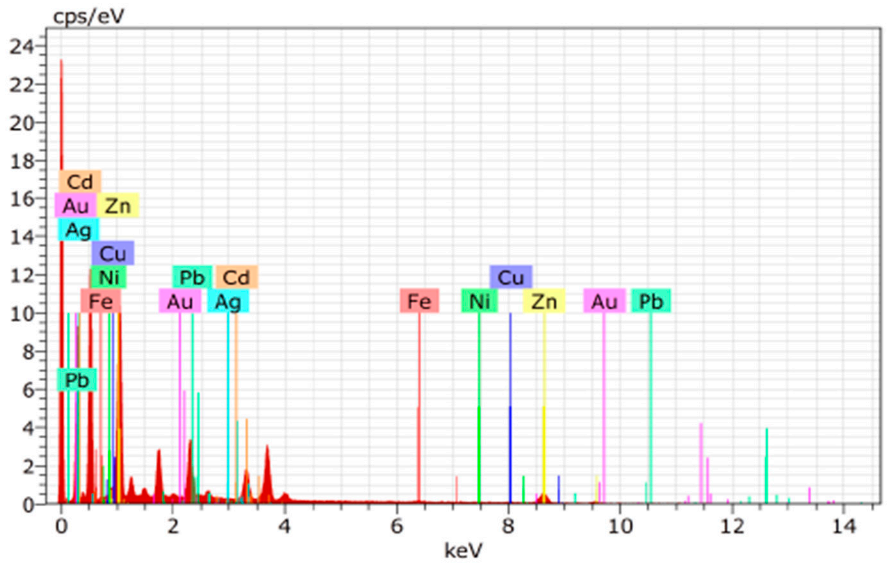

Figure S2. ESEM micrograph and EDS analysis of flocs obtained with CA at 800 mg·L^{-1} (FA800). Yellow arrows indicate selected inorganic domains.

FB400

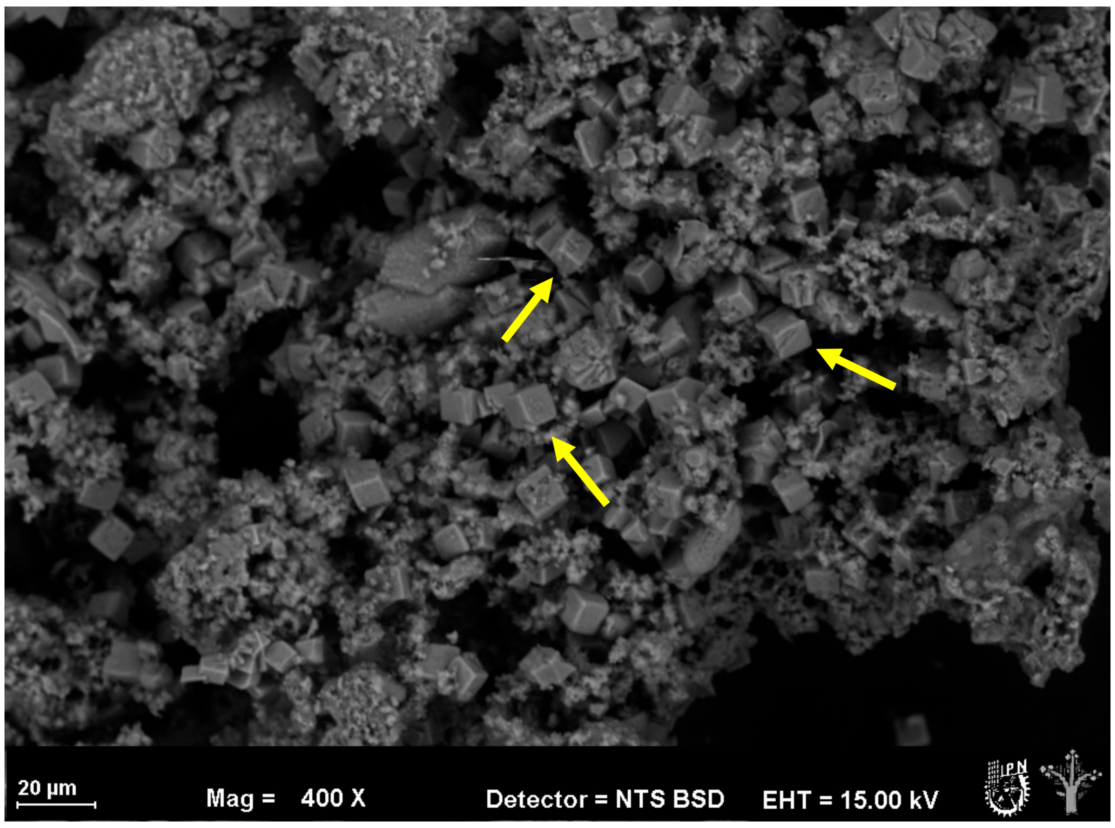

Spectrum: Acquisition 2912

Element	Atom. C [at.%]
Iron	3.36
Manganese	0.00
Nickel	0.00
Copper	5.72
Silver	0.00
Gold	2.11
Zinc	39.49
Cadmium	28.16
Lead	21.17
Total:	100.00

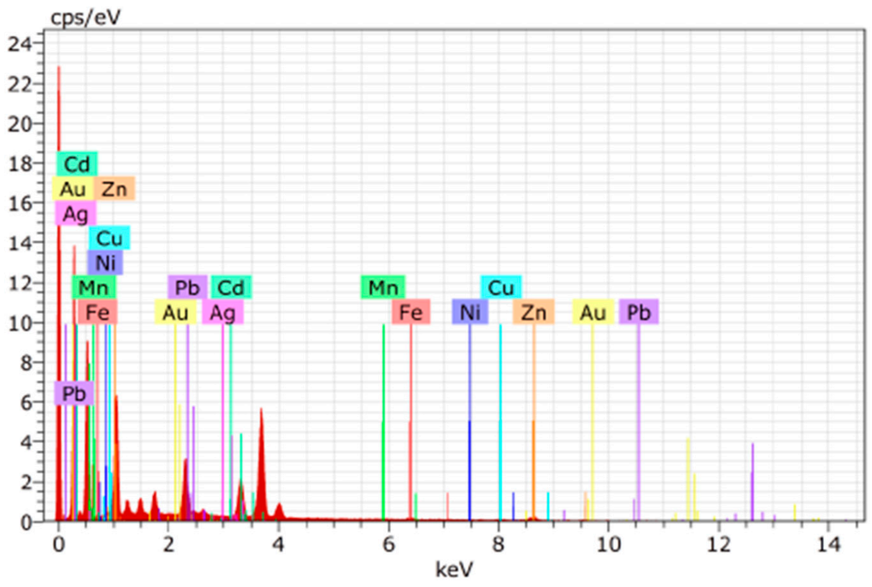

Figure S3. ESEM micrograph and EDS analysis of flocs obtained with CB at 400 mg·L^{-1} (FB400). Yellow arrows indicate selected inorganic domains analyzed.

FB800

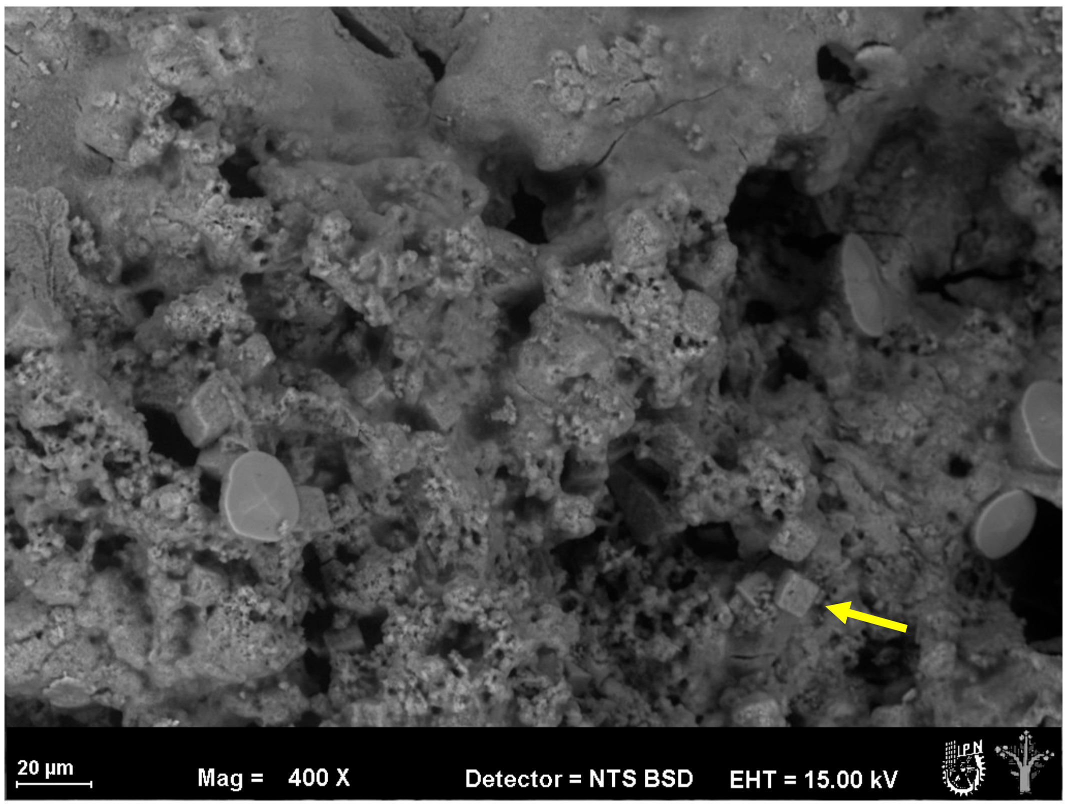

Spectrum: Acquisition 2914

Element	Atom. C [at.%]
Iron	2.35
Manganese	0.30
Nickel	0.67
Copper	10.12
Silver	0.00
Gold	1.29
Zinc	50.55
Lead	14.70
Cadmium	20.03
Total:	100.00

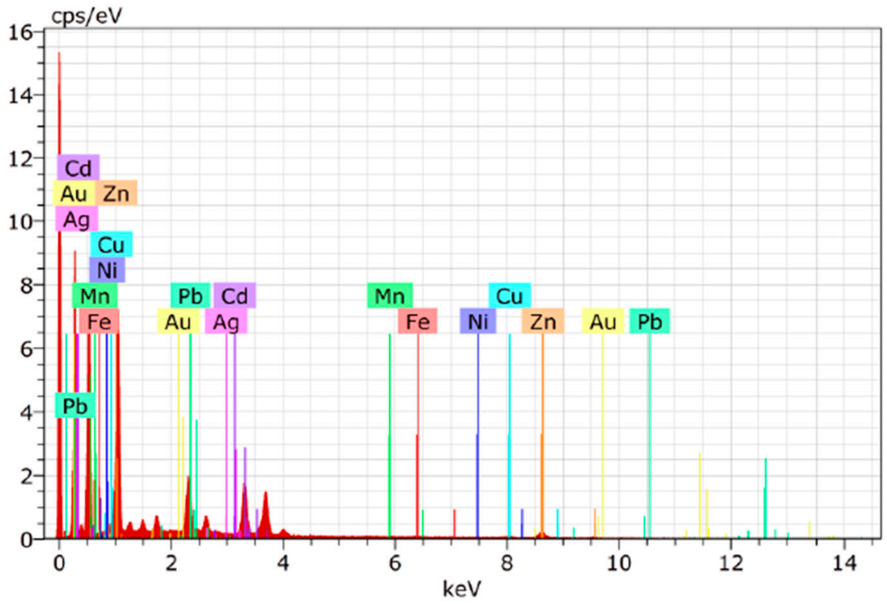

Figure S4. ESEM micrograph and EDS analysis of flocs obtained with CB at 800 mg·L^{-1} (FB800). Yellow arrows indicate selected inorganic domains.

FC400

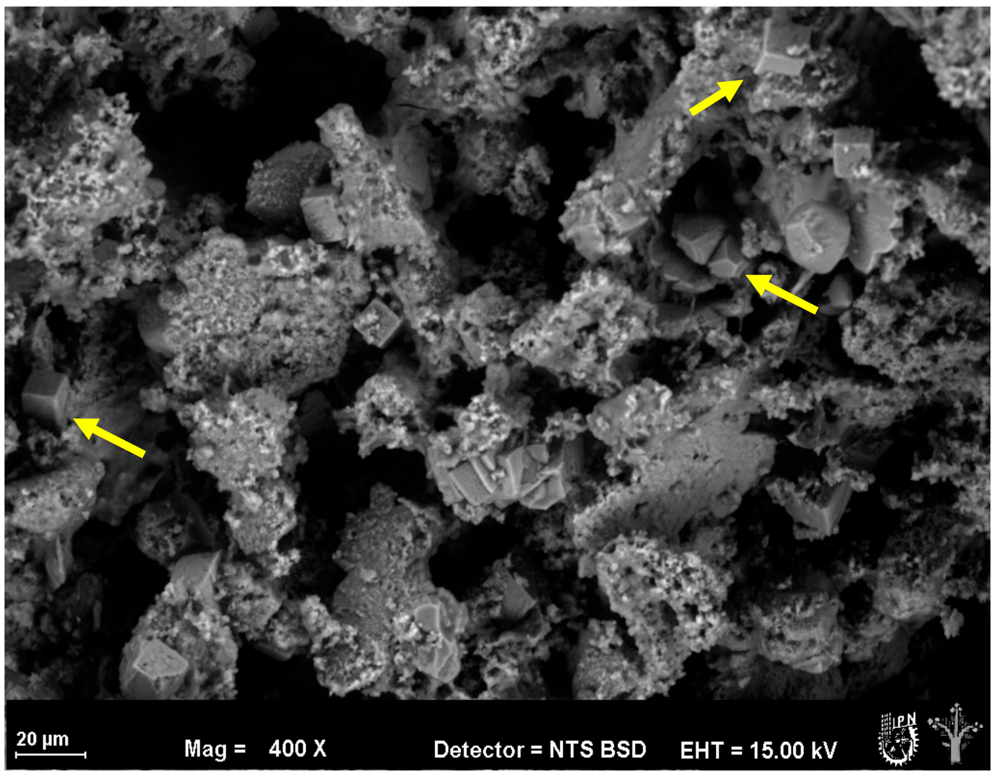

Spectrum: Acquisition 2924

Element	Atom. C [at.%]
Manganese	0.28
Iron	4.52
Nickel	0.50
Copper	8.12
Silver	0.00
Gold	1.10
Zinc	48.89
Cadmium	21.56
Lead	15.04
Total:	100.00

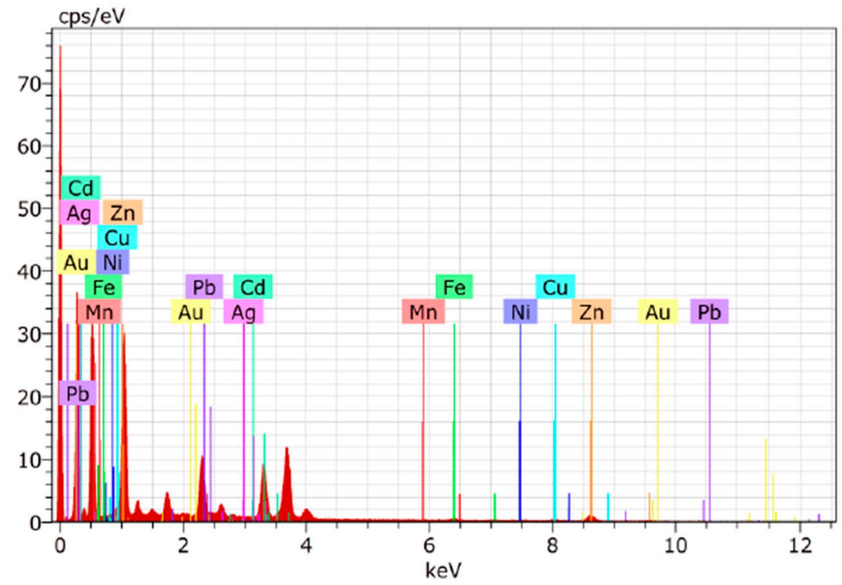

Figure S5. ESEM micrograph and EDS analysis of flocs obtained with CC at 400 mg·L^{-1} (FC400). Yellow arrows indicate selected inorganic domains.

FC800

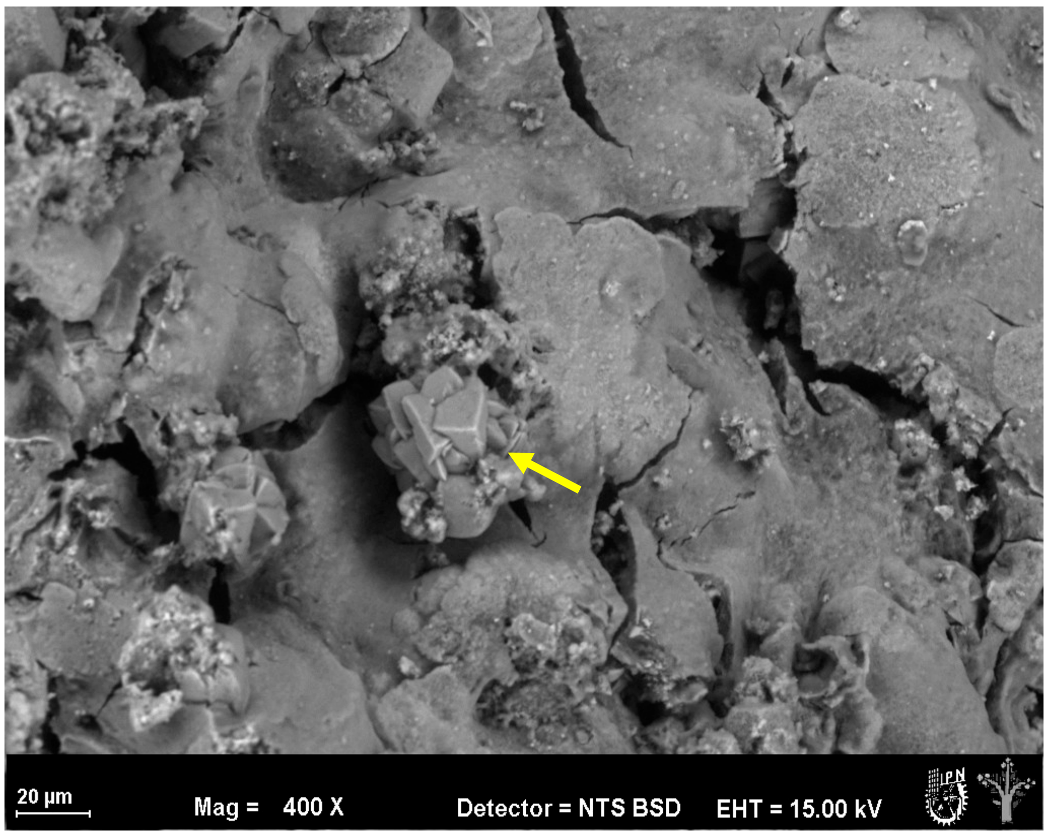

Spectrum: Acquisition 2926

Element	Atom. C [at.%]
Manganese	0.00
Iron	4.72
Nickel	0.39
Copper	6.33
Silver	0.00
Gold	1.97
Zinc	46.79
Cadmium	23.68
Lead	16.12
Total:	100.00

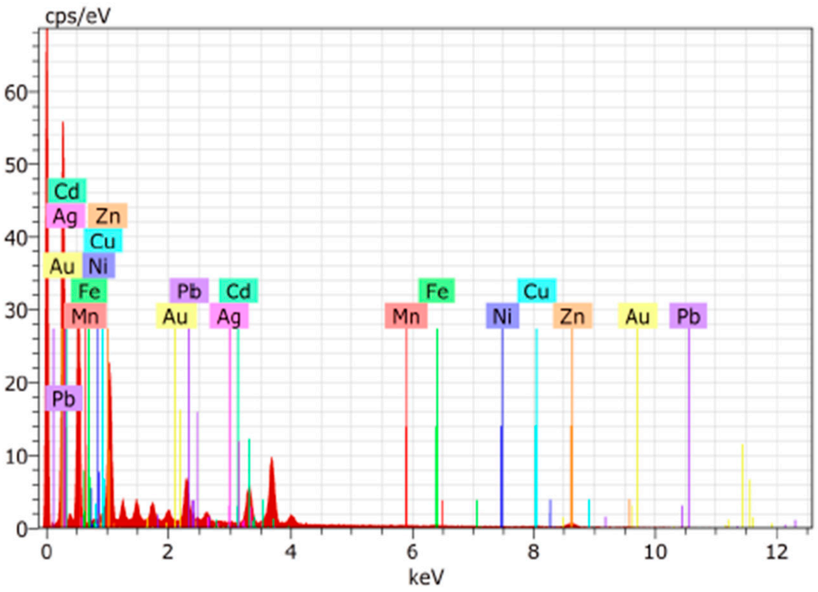

Figure S6. ESEM micrograph and EDS analysis of flocs obtained with CC at 800 mg·L^{-1} (FC800). Yellow arrows indicate selected inorganic domains.
